# Vertigoheel promotes rodent cognitive performance in multiple memory tests

**DOI:** 10.3389/fnins.2023.1183023

**Published:** 2023-05-31

**Authors:** Kerstin Ott, Taneli Heikkinen, Kimmo K. Lehtimäki, Kaisa Paldanius, Jukka Puoliväli, Raimo Pussinen, Emile Andriambeloson, Bertrand Huyard, Stéphanie Wagner, Cathrin Schnack, Anke Wahler, Bjoern von Einem, Christine A. F. von Arnim, Yvonne Burmeister, Kathrin Weyer, Bernd Seilheimer

**Affiliations:** ^1^Heel GmbH, Baden-Baden, Germany; ^2^Charles River Discovery Services, Kuopio, Finland; ^3^Neurofit SAS, Illkirch, France; ^4^Department of Neurology, Ulm University, Ulm, Germany; ^5^Department of Geriatrics, University Medical Center Göttingen, Göttingen, Germany

**Keywords:** Vertigoheel, visual recognition memory, spatial working memory, spatial orientation memory, multicomponent drug, olfactory memory, forgetfulness, neurite length

## Abstract

**Introduction:**

Cognitive impairment associated with old age or various brain disorders may be very disabling for affected individuals, placing their carers and public health services under considerable stress. The standard-of-care drugs produce only transient improvement of cognitive impairment in older people, so the search for novel, safe and effective therapeutics that would help to reverse or delay cognitive impairment is warranted. Repurposing pharmacological therapies with well-established safety record for additional indications is a promising recent trend in drug development. Vertigoheel (VH-04), a multicomponent drug made of *Ambra grisea*, *Anamirta cocculus L.*, *Conium maculatum*, and *Petroleum rectificatum*, has been successfully used for several decades in the treatment of vertigo. Here, we investigated effects of VH-04 on cognitive performance in standard behavioral tests assessing different types of memory and explored cellular and molecular underpinnings of VH-04’s biological activity.

**Methods:**

In the majority of behavioral experiments, namely in the spontaneous and rewarded alternation tests, passive avoidance test, contextual/cued fear conditioning, and social transmission of food preference, we examined the ability of single and repeated intraperitoneal administrations of VH-04 to improve cognitive parameters of mice and rats disrupted by the application of the muscarinic antagonist scopolamine. In addition, we also assessed how VH-04 affected novel object recognition and influenced performance of aged animals in Morris water maze. Furthermore, we also studied the effects of VH-04 on primary hippocampal neurons *in vitro* and mRNA expression of synaptophysin in the hippocampus.

**Results:**

Administration of VH-04 positively influenced visual recognition memory in the novel object recognition test and alleviated the impairments in spatial working memory and olfactory memory caused by the muscarinic antagonist scopolamine in the spontaneous alternation and social transmission of food preference tests. In addition, VH-04 improved retention of the spatial orientation memory of old rats in the Morris water maze. In contrast, VH-04 did not have significant effects on scopolamine-induced impairments in tests of fear-aggravated memory or rewarded alternation. Experiments *in vitro* showed that VH-04 stimulated neurite growth and possibly reversed the age-dependent decrease in hippocampal synaptophysin mRNA expression, which implies that VH-04 may preserve synaptic integrity in the aging brain.

**Discussion:**

Our findings allow a cautious conclusion that in addition to its ability to alleviate manifestations of vertigo, VH-04 may be also used as a cognitive enhancer.

## 1. Introduction

Cognitive impairment (CI) associated with old age places a heavy burden on the affected individuals, public health services and the society as a whole. Mild cognitive impairment (MCI) prevalence is 1–3% in the total population, rising to 25% in 80–84-year-old individuals (Fisk et al., 2003; Petersen et al., 2018). MCI is generally considered a predictor of dementia occurrence, and the incidence of certain forms of MCI may be increasing in some countries (Stephan et al., 2022). It is generally accepted that the standard-of-care drugs, such as the acetylcholinesterase inhibitor donepezil and *N*-methyl-D-aspartate glutamate receptor antagonist memantine, produce only transient improvement of CI in older people (Gauthier and Touchon, 2005; Patnode et al., 2020). Therefore, there is a considerable unmet need for safe and effective therapies for CI.

Contemporary drug development is a costly and protracted process, so drug repurposing may be a promising strategy for cognitive enhancement. For example, a third of the drugs in the current Alzheimer’s disease development pipeline are repurposed (Cummings et al., 2022). Further, because CI has multiple causes and involves dysfunction of different receptors and signaling cascades, novel molecular entities developed by single-target screening often have suboptimal efficacy in disorders of cognition. In this regard, multicomponent formulations may offer a possibility of engaging several molecular targets simultaneously (Keith et al., 2005).

Vertigoheel, also known as VH-04, is a multicomponent, multitarget drug made of *Ambra grisea*, *Anamirta cocculus L.*, *Conium maculatum*, and *Petroleum rectificatum*. According to several clinical studies, VH-04 significantly reduces the frequency, duration and intensity of vertigo (Weiser et al., 1998; Wolschner et al., 2001; Issing et al., 2005; Schneider et al., 2005). Beneficial effects of VH-04 may be mediated by the vasodilatory or neurotropic actions of the drug (Klopp et al., 2005; Heinle et al., 2010; Dimpfel et al., 2019). In our recent experiments in a rat model of vestibular syndrome, we noted that VH-04 seemed to improve performance in the eight-arm radial maze task in sham-treated animals (Hatat et al., 2022). We therefore sought to investigate effects of VH-04 in several preclinical memory tests to assess its potential as a cognitive enhancer. To this end, we examined how intraperitoneal injections of VH-04 influenced visual recognition memory, spatial working memory, spatial orientation learning and memory, fear-aggravated memory and olfactory memory in rats and mice. In addition, to explore the molecular and cellular underpinnings of the biological activity of VH-04, we examined the levels of hippocampal metabolites in VH-04-treated animals and assessed the effect of the drug on the mRNA expression of the synaptic marker synaptophysin in the hippocampal tissue of aged rats and on the neurite length in cultured mouse primary neurons.

## 2. Materials and methods

### 2.1. Study locations

**Figure 1:**
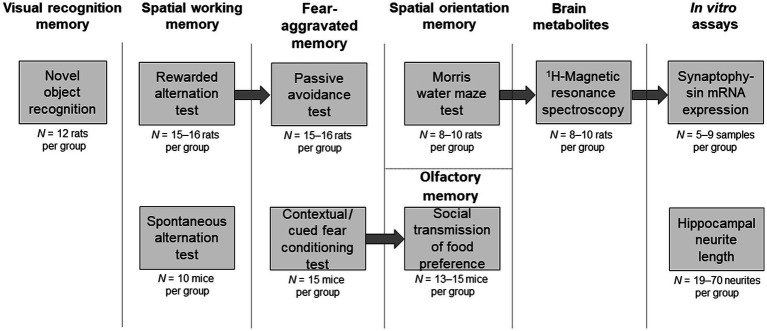
Schematic illustration of the experiments. Behavioral tests or *in vitro* assays are described in individual boxes. Arrows connect experiments where the same set of animals was used following breaks of several weeks to allow wash-out of the drugs from the previous experiment. Upper row of boxes illustrates experiments in rats or involving rat tissue. Lower row of boxes illustrates experiments in mice or involving mouse tissue. The initial number of animals allocated per group is indicated under each box. Occasionally, data from some animals were excluded (see section 2.3 Behavioral tests for details and justification of such exclusions). Headings above the boxes indicate the cognitive domain or other experimental parameter tested.

Experiments evaluating *in vivo* and *in vitro* effects of VH-04 are summarized in Figure 1. These experiments were performed at three different locations as follows. Novel object recognition and spontaneous alternation in the T-maze tests were carried out by Neurofit S.A.S. in accordance with French Animal Health Regulations (Permit No. D 67–218-23 issued by the “Direction Départementale des Services Vétérinaires” of the Ministry of Agriculture and Fisheries.) All other *in vivo* experiments, including rewarded alternation in the T-maze, Morris water maze, passive avoidance, fear conditioning and social transmission of food preference tests, were performed by Charles River Discovery Services. These experiments were carried out according to the National Institute of Health (NIH) guidelines for the care and use of laboratory animals and approved by the State Provincial Office of Southern Finland (license numbers 605/01/2006 and ESLH-2008-04752/Ym-23). Hippocampal metabolic profiling and determination of synaptophysin mRNA levels in hippocampal samples of young and old VH-04-treated rats was done at Charles River Discovery Services. Experiments in cultured mouse primary hippocampal neurons were conducted in the Department of Neurology at the Ulm University.

### 2.2. Drugs

The VH-04 solution contained 70 μg/mL extract of dried, ripe fruit of *Anamirta cocculus* L., 20 μg/mL extract of fresh aerial parts of flowering (but not fruiting) *Conium maculatum* plants, 10^−4^ μg/mL *Petroleum rectificatum* obtained from crude oil by distilling between 180 and 220°C and 10^−2^ μg/mL of *Ambra grisea* excreted from the intestines of the sperm whale *Physeter catodon L.* The VH-04 solution was manufactured and bottled in 1.1 mL glass ampoules by Heel GmbH (Baden-Baden, Germany) according to the international Good Manufacturing Practice standards. Vehicle control contained 0.9% NaCl (Sigma Aldrich, St. Louis, MO, United States or Heel GmbH, Baden-Baden, Germany) in sterile water. In experiments *in vivo*, VH-04 was administered intraperitoneally to experimental animals at doses of 0.1–2 mL/kg once or on multiple occasions, depending on the task-specific treatment regimen. The dilutions of the original VH-04 solution were done using 0.9% NaCl solution. For experiments *in vitro*, the original VH-04 solution or vehicle was diluted with culture medium at the following ratios (culture medium:VH-04 or vehicle): 1:3, 1:4, and 3:1.

The nonselective muscarinic antagonist scopolamine hydrobromide (cat. #S0929, Sigma-Aldrich, St. Louis, MO, United States) was used to induce CI in mice and rats following intraperitoneal administration at a dose of 1 mg/kg (dosage volume 10 mL/kg or 2 mL/kg for passive avoidance and rewarded alternation tests). The clinically active cholinesterase inhibitor donepezil hydrochloride (cat. # D6821, Sigma-Aldrich, St. Louis, MO, United States) was used as positive control to ameliorate the effects of scopolamine. Donepezil was administered intraperitoneally at a dose of 0.3 mg/kg (dosage volume 2 mL/kg in the novel object recognition test or 10 mL/kg in the spontaneous alternation test) or 1 mg/kg (dosage volume 10 mL/kg for contextual/cued fear conditioning, and social transmission of food preference tests or 2 mL/kg for passive avoidance, and rewarded alternation tests).

### 2.3. Behavioral tests

#### 2.3.1. Novel object recognition

The novel object recognition (NOR) test was performed in 72 male Wistar rats (Janvier, Genest-Saint-Isle, France) that were group-housed at a density of 12–14 animals per cage and maintained in a room with controlled temperature (21–22°C) with relative humidity range of 26–41%, The room was kept on a reversed light–dark cycle (12 h/12 h, lights on: 17:30–05:30; lights off: 05:30–17:30). Food (A04; Safe, Epinay-sur-Orge, France) and water were available *ad libitum*.

Experiments were performed in an open field acrylic glass arena (100 cm × 100 cm with 45 cm walls) within which animals moved freely. The two objects used in the experiments were a metallic ball and a black box, which had similar volume. Approaches to the objects were recorded by a video tracking system (Viewpoint, France).

The day before testing, the rats were allowed to explore the open field arena during a 15 min habituation session. The next day, object A, named “familiar object,” was placed at the periphery of a virtual central square (30 cm × 30 cm) of the open field. Rats were allowed to interact with this object for 10 min (acquisition trial). Animals that did not display locomotor activity (total immobility) or failed to explore the object were excluded. After a 30 min (group 1, see below) or a 24 h delay (other five groups) to induce natural forgetting (Roncarati et al., 2009), object B (“novel object”) was placed adjacent to object A which remained in the same location as during the acquisition trial. Animals were left to explore both objects for 10 min. The times spent exploring each of the two objects during the retention trial (*t*_A_ and *t*_B_, respectively) were recorded separately. A rat was considered to explore the object if its nose was directed to it within a distance of ≤2 cm. Animals that were totally immobile or failed to explore the objects for longer than 5 s in total (*t*_A_ + *t*_B_) were excluded.

Rats were randomly assigned to one of the six different experimental groups (*N* = 12 per group): (1) 30 min/saline; (2) 24 h/saline; (3) 24 h/0.1 mL/kg VH-04; (4) 24 h/1 mL/kg VH-04; (5) 24 h/2 mL/kg VH-04; and (6) 24 h/0.3 mg/kg donepezil. Each animal was identified by its group name, cage number, series (day) of experiment, and the number (1–12) written with permanent ink on the tail. The performance of rats from group 1, where the retention trial was performed in 30 min was considered as the optimal level of recognition as the spontaneous decay of memory was not present yet.

The original preparation of VH-04 was administered intraperitoneally at doses of 0.1, 1, and 2 mL/kg. The cholinesterase inhibitor donepezil was given intraperitoneally at a dose of 0.3 mg/kg. The dosage volume for both VH-04 and donepezil was 2 mL/kg, and lower doses were obtained by dilution with 0.9% NaCl. VH-04 and donepezil were administered 40 and 30 min before the acquisition trial, respectively.

Visual recognition memory was evaluated using the discrimination index (DI_time_) that corresponds to the proportion (%) of time spent investigating object B during the retention trial with respect to the total time spent investigating both familiar object A and new object B. DI_time_ was calculated as follows: DI_time_ = *t*_B_/(*t*_A_ + *t*_B_) × 100%, where *t* denotes time. In addition, we also calculated DI_contacts_ calculated as the ratio of number of contacts with the novel object divided by the total number of contacts with both familiar and novel objects: DI_contacts_ = *n*_B_/(*n*_A_ + *n*_B_) × 100%, where *n* denotes the number of contacts.

Two rats from the group 30 min/saline and one rat from the group 24 h/0.1 mL/kg VH-04 were excluded from the analysis as they explored the objects for less than 5 s in total during the retention trial.

#### 2.3.2. T-maze spontaneous alternation

The T-maze spontaneous alternation test was performed in 4–5-week-old male CD-1 mice (Janvier, Genest-Saint-Isle, France). The animals were group-housed (10 mice per cage) and maintained in a room with controlled temperature (21–22°C) and reversed light–dark cycle (12 h/12 h; lights on: 17:30–05:30; lights off: 05:30–17:30). Food and water were available *ad libitum*.

Experiments were performed in a T-maze made of gray Plexiglas with a main stem (55 cm long × 10 cm wide × 20 cm high) and two goal arms (30 cm long × 10 cm wide × 20 cm high) positioned at a 90° angle relative to the main stem. The start box (15 cm long × 10 cm wide) was separated from the main stem by a sliding door. Sliding doors were also used to close specific arms during the force choice alternation trial. Each mouse was tested in single session that started with a “forced-choice” trial and was followed by 14 “free-choice” trials. In the first “forced-choice” trial, the animal was confined for 5 s in the start box and then released while either the left or right goal arm was blocked by a horizontal door. After the animal eventually entered the open goal arm, it spontaneously returned to the start box. Immediately thereafter, the left and right goal arm doors were opened, and the animal was allowed to choose freely between the left and right goal arm (“free choice trials). The animal was considered to have entered an arm when all its four paws were in the arm. The session was terminated and the animal was removed from the maze as soon as 14 free-choice trials had been completed or after 10 min had elapsed, whatever occurred first. Urine and feces were removed, and the maze was cleaned between each animal using a 70% ethanol solution. During the trials, animal handling and the visibility of the operator were minimized as much as possible.

Mice were randomly assigned to one of the seven experimental groups to examine whether single or repeated treatment with VH-04 could reverse the amnesic effect of the nonselective muscarinic antagonist scopolamine. For single treatment, VH-04 or vehicle (0.9% NaCl) were administered intraperitoneally 40 min before the T-maze trial. For repeated treatment, VH-04 was administered once or twice a day for 3 days, with the last injection performed 40 min before the T-maze trial. Scopolamine (1 mg/kg) or its vehicle (0.9% NaCl) was injected intraperitoneally 20 min before the T-maze trial. In one group, scopolamine was co-injected with 0.3 mg/kg donepezil. Thus, the animals in the experimental groups received the following injections (*N* = 10 per group): (1) vehicle_VH-04_ and vehicle_scopolamine_; (2) vehicle_VH-04_ and scopolamine; (3) single 1 mL/kg VH-04 and scopolamine; (4) single 2 mL/kg VH-04 and scopolamine; (5) repeated, once daily 1 mL/kg VH-04 for 3 days and scopolamine; (6) repeated, twice daily 1 mL/kg VH-04 for 3 days and scopolamine; and (7) donepezil and scopolamine. Spontaneous alternations were counted as entries into a T-maze arm different from that visited in the previous trial. The percentage of spontaneous alternations over the 14 free-choice trials was calculated as an index of working memory performance.

#### 2.3.3. T-maze rewarded alternation

Ninety-five adult male Wistar rats (Charles River, Germany) weighing 225–350 g were used for the T-maze rewarded alternation experiments. Animals were housed at 22 ± 1°C in a light-controlled environment (lights on: 7:00–20:00; lights off: 20:00–7:00). Food and water were supplied *ad libitum* during maintenance and habituation period, but during T-maze training and testing, rats were kept at 80% of their free-feeding body weight by restricting their free access to food to 1–1.5 h daily.

Behavioral testing was conducted in a T-maze with black walls and floors. The start box (10 cm wide × 20 cm long × 15.5 cm high) was separated from the start arm (10 cm wide × 64 cm long × 15.5 cm high) by an opening and a manually operated guillotine door. The start arm terminated in a choice point (10 cm wide × 27 cm long ×15.5 cm high). The two goal arms (10 cm wide × 75 cm long × 15.5 cm high) were separated from the choice point by openings and guillotine doors that prevented animals from re-entering the choice point. A goal box (10 cm wide × 20 cm long × 15.5 cm high) at the end of each goal arm contained a black plastic dish in which the food reward was placed. The maze was located in the rat colony room, and a variety of fixed spatial cues were placed around the apparatus.

During the task, each animal received a food reward (Kellogg’s Froot Loop) on the first trial regardless of which goal box was entered. On each subsequent trial, the animal was required to visit the arm not chosen on the previous trial to obtain food reward. All rats received one set of five alternation trials daily, and incomplete trials were terminated after 2 min. The intertrial interval was approximately 15 s, i.e., the amount of time required to transfer an animal from the goal box to the start box. This training period ended after 12 days.

As in the spontaneous alternation test described in section 2.3.2., the effects of VH-04 on cognition were assessed based on its ability to rescue T-maze performance after scopolamine injections. For single treatment, VH-04 was administered intraperitoneally together with 1 mg/kg scopolamine 30 min before the T-maze test trial. For repeated treatment, VH-04 or vehicle_VH-04_ was administered once daily for 3 days, and the last injection was performed together with 1 mg/kg scopolamine or its vehicle 30 min before the T-maze test. In one group, scopolamine was co-injected with 1 mg/kg donepezil. Dosing volume was 2 mL/kg for all treatments. There were six experimental groups, in which animals received the following treatments: (1) vehicle_VH-04_ once daily for 3 days and vehicle_scopolamine_ 30 min prior to T-maze test (*N* = 15); (2) vehicle_VH-04_ once daily for 3 days and scopolamine (*N* = 16); (3) single 1 mL/kg VH-04 and scopolamine (*N* = 16); (4) single 2 mL/kg VH-04 and scopolamine (*N* = 16); (5) repeated, once daily 1 mL/kg VH-04 for 3 days and scopolamine (*N* = 16); (6) donepezil and scopolamine (*N* = 16). The percentage of correct alternations on the test day was calculated. Rats that completed fewer than three out of five alternation trials on the test day were excluded from analysis. Based on the exclusion criteria, data from 15, 9, 10, 9, 9, and 12 animals were included into final analysis for groups 1–6, respectively.

#### 2.3.4. Passive avoidance

The passive avoidance test was performed in the same cohort of Wistar rats that was used for the rewarded alternation test (see section 2.3.3). The animals were randomly re-grouped after a period of several days to allow washout of previously administered drugs. Data from one out of the 95 rats were excluded from analysis due to technical fault of the equipment.

Passive avoidance behavior was tested in a Gemini Avoidance system (San Diego Instruments, San Diego, CA, United States). Rats received a single training trial followed by a test trial 24 h later. For the acquisition trial, each rat was placed in the start chamber for a 15 s adaptation period, after which the start chamber was illuminated, and a guillotine door opened exposing a dark chamber. When the rat entered the dark side, the guillotine door was closed and the animal received a 0.4 mA foot shock for 3 s. Time to enter the dark chamber was recorded. The retention trial performed 24 h later was identical to the acquisition one except that if the rat entered the dark chamber the guillotine door was closed and no shock was delivered. The acquisition and retention trials had a maximum duration of 600 s. Escape latency values from the acquisition and retention trials were used in statistical analyses. Dosing volumes and doses of drugs used in the six experimental groups were similar to those during the T-maze rewarded alternation test except for alterations in VH-04 treatment regimens: (1) vehicle_VH-04_ once daily for 3 days and vehicle_scopolamine_ 30 min prior to T-maze test (*N* = 16); (2) vehicle_VH-04_ once daily for 3 days and scopolamine (*N* = 16); (3) single 2 mL/kg VH-04 and scopolamine (*N* = 15); (4) repeated, once daily 1 mL/kg VH-04 for 3 days and scopolamine (*N* = 15); (5) repeated, once daily 2 mL/kg VH-04 for 3 days and scopolamine (*N* = 16); (6) donepezil and scopolamine (*N* = 16).

#### 2.3.5. Contextual/cued fear conditioning

Ninety male 12-week-old C57Bl/6 J mice (Charles River, Sulzfeld, Germany) were used in fear conditioning experiment. Animals were housed at 22 ± 1°C in a light-controlled environment (lights on: 7:00–20:00; lights off: 20:00–7:00). Food and water were supplied *ad libitum*.

Fear conditioning training and testing were conducted on two consecutive days using a Coulbourn FreezeFrame system (Coulbourn, Whitehall, PA, United States). On day 1, the mouse was placed into the chamber with a bright house light on and allowed to explore for 2 min. Then, an auditory cue (1,700 Hz, 80 dB; conditioned stimulus) was presented for 15 s. A 2 s electric foot shock (1.5 mA; unconditioned stimulus) was administered for the final 2 s of the conditioned stimulus. This procedure was repeated, and after another 30 s, the mouse was removed from the chamber. After 20 h, on test day 2, the mouse was returned to the same chamber in which the training occurred, and its freezing behavior was recorded by the software (memory for context). Freezing was defined as episodes of the lack of movement, except for that required for respiration, which lasted for at least 2 s. At the end of the 5 min context trial, the mouse was returned to its home cage. One hour later, the mouse was placed in another chamber with lower lighting level and wall and floor materials different from those in the original chamber. Freezing behavior in this novel environment (altered context) was recorded for 3 min. Then auditory cue (conditioned stimulus) was presented for 3 min and freezing behavior was analyzed again (memory for cue). Freezing scores for each subject were expressed as a percentage of time spent immobile during each part of the test on day 2 (memory for context, altered context, memory for cue).

Drug concentrations and timings of drug treatments with respect to testing were identical to those used in T-maze rewarded alternation experiments (see section 2.3.3) except that the dosing volume was 10 mL/kg. There were 15 mice per group initially. One mouse from the group SCO + VH-04 (1 mL/kg, q.d., 3 days) and one mouse from the group SCO + donepezil (1 mg/kg) had to be terminated due to severe fighting wounds. Another mouse from the group SCO + VH-04 (1 mL/kg, q.d., 3 days) was excluded from analysis because of an incorrect injection. Thus, groups 1–6 comprised 15, 15, 15, 15, 13, and 14 animals, respectively.

#### 2.3.6. Social transmission of food preference

The social transmission of food preference (STFP) test was performed in the same cohort of C57Bl/6 J mice that was used for fear conditioning experiments (see section 2.3.5). Drug concentrations and timings of drug treatments with respect to testing were identical to those used in T-maze rewarded alternation experiments (see section 2.3.3) except that the dosing volume was 10 mL/kg. The animals were randomly re-grouped after a period of 4 weeks to allow washout of previously administered drugs during fear conditioning testing. In addition to the mice removed from this cohort during contextual/cued fear conditioning, one mouse from the group SCO + VH-04 (1 mL/kg) and two mice from the group SCO + VH-04 (2 mL/kg) had to be terminated due to severe fighting wounds. Another mouse from the group SCO + VH-04 (1 mL/kg) was excluded from analysis because of an incorrect injection. Thus, groups 1–6 in this experiment comprised 15, 14, 13, 13, 14, and 15 animals, respectively.

The protocol for the STFP test was a slightly modified version of the test described by Wrenn (2004). The STFP test involves two socially familiar mice, one designated observer and the other demonstrator. The mice were group-housed at a ratio of no greater than four observer mice to one demonstrator mouse. Before the experiment, all animals had unlimited access to ground chow and water. At the end of the group-feeding phase, the ground chow was removed from the cage and replaced by two cups of powdered chow that were placed in the home cages, so that the mice were habituated to eat powdered chow from the cups. This habituation phase lasted 4 days. After the habituation phase, on day 0, the demonstrator mouse was removed from the cage and single housed without access to food. The demonstrator mice were not included in any of the treatment groups throughout the experiment. On the next day (day 1), the cages of the observer mice were changed to clean ones, and all food was removed from the cages. This took place 4 h before the scheduled interaction with the demonstrator mouse. After a 24 h food deprivation period, each demonstrator mouse was placed in an empty cage with a cup of powdered food, scented either with cinnamon (1% w/w) or cocoa (2% w/w). All the mice and groups were divided equally for exposure to these two flavors. Each demonstrator mouse was given 1.5 h to eat the scented food. Immediately after this, they were placed back in their original cages with the observer mice and allowed to interact with their respective observer cage mates for 30 min. Demonstrators were then removed and observer mice left undisturbed for 24 h. After that period, on day 2, each observer mouse was placed in a unique test cage and allowed to eat for 1.5 h. Test cages contained two pre-weighed cups, one with cinnamon-flavored chow and another - with cocoa flavored chow. The scent presented by the demonstrator mouse 24 h earlier was the “cued food” and the other, novel scent was the “non-cued food.” The location of the novel food in the cage (front or rear end of the cage) was counterbalanced across mice. After 1.5 h, observer mice were returned to their home cages and the cups were weighed again to determine the consumed amounts of the “cued” and “non-cued” food.

#### 2.3.7. Morris water maze

Spatial orientation learning and memory in Morris water maze (MWM) were studied in 16 mature adult male Sprague Dawley rats aged 25 months (Harlan, Indianapolis, IN, United States) and 10 young adult male Sprague Dawley rats aged 3 months (CRL, Sulzfeld, Germany), weighing ~600 and 300 g, respectively. Animals were housed at 22 ± 1°C in a light-controlled environment (lights on: 7:00–20:00; lights off: 20:00–7:00). Food and water were supplied *ad libitum.* The animals were divided into three experimental groups that received either vehicle (0.9% NaCl) or VH-04 intraperitoneally at a dose of 2 mL/kg once a day for 14 days before the first day of the MWM test and then once daily during testing until termination as follows: (1) eight mature rats, vehicle; (2) eight mature rats, VH-04; (3) 10 young rats, vehicle. On the days of behavior testing, the treatment was administered 30 min before the test.

Testing was performed in a large dark-colored circular tank (200 cm in diameter) filled with clear water at a temperature of 21.0 ± 1.0°C. The rim of the tank had eight starting point labels situated at equal distances: N, NE, E, SE, S, SW, W, and NW. A submerged 10 × 10 cm square platform was placed in the middle of the quadrant between S and W at 1.5 cm below water surface. The rats were lowered into the pool with their nose pointing toward the wall at any of the starting points except SW, as it was close to platform location.

On testing day 0, rats received visible platform pre-training to determine whether any non-cognitive performance deficits (e.g., visual impairments and/or swimming difficulties) were present, which could affect performance during subsequent acquisition and probe trials. All rats received four trials with inter-trial intervals of 15 min on testing day 0. During each pre-training trial, rats were placed in a fixed position in the swimming pool facing the wall and allowed to swim to a platform, which was randomly placed in any of the 4 quadrants of the tank and marked with a rod (visual cue) that extended 20 cm above water level. Rats were given 60 s to find the platform, which they stayed on for 15 s before being removed from the pool. If a rat did not find the platform within 60 s, it was gently guided to the platform and allowed to remain there for 15 s. The time for each rat to reach the cued platform, thigmotaxis, and the swim speed were recorded. After the visible platform pre-training was completed, the data was analyzed and the rats were assigned to the different treatment groups based on their pre-training performance. This procedure was performed to ensure that each treatment group comprised equal numbers of good and poor performers in the cued version of the MWM task.

In 14 days after the completion of cued trials, rats received acquisition (place) trials to determine their ability to learn the spatial relationship between distant cues and the escape platform, which remained in the same location (SW) during all place trials, but was submerged and without visual cue. The starting points were randomized and starting point SW was not used. The time to reach the platform was recorded for each trial. The less time it took a rat to reach the platform, the better the learning ability was. The rats received acquisition testing on 9 separate days with four place trials (15 min apart, 60 s maximum for each trial) on each of those days. There was a 3-day break between test days 4 and 5. In addition to latency, path length, thigmotaxis and swim speed were recorded. Following the last day of acquisition trials, rats had a 1-day break, and a single probe trial was conducted to evaluate memory retention capabilities. During the probe trial, the platform was removed from the MWM pool, and each rat was placed into the MWM pool in quadrant NE, opposite to one where the platform was before. The rats were allowed to swim for 60 s during the probe trial. The time spent in the target quadrant and target platform annulus (36-cm-diameter circular area surrounding platform) as well as and crosses over the target platform position were recorded to assess the extent of memory retention.

### 2.4. ^1^H-magnetic resonance spectroscopy

^1^H-Magnetic Resonance Spectroscopy (^1^H-MRS) was performed in young and aged rats from the MWM experimental cohort in 1–4 days following probe trials (6–7 rats per day). MR acquisition was performed in a horizontal 7 T magnet with a bore size of 160 mm (Magnex Scientific Ltd., Oxford, United Kingdom). The system was equipped with a Magnex gradient set (max. Gradient strength 400 mT/m, bore 100 mm) interfaced to a Varian DirectDrive console (Varian, Inc., Palo Alto, CA, United States) using a 63 mm diameter volume coil for transmission and surface phased array coil for receiving (Rapid Biomedical GmbH, Rimpar, Germany). Rats were fixed to a head holder with ear-pins and tooth-bar (Rapid Biomedical GmbH) and positioned in the magnet bore in a standard orientation relative to gradient coils. Rectal temperature and respiration were monitored throughout the study and levels were kept at 36–37°C and 45–60 breaths/min, respectively. After animal positioning (fast low angle shot gradient echo sequence scout images; axial, coronal and sagittal), T2-weighted multi-slice images (10 continuous slices) were acquired using fast spin-echo sequence with TR/TEeff = 1,500/48 ms, matrix size of 256 × 128, field of view of 35 × 35 mm^2^, slice thickness of 0.5 mm, and two averages.

^1^H-MRS data were collected using the same experimental setup. Bilateral voxel of 8 × 2 × 4 mm^3^ was placed in rat hippocampus based on the T2-weighted images collected as described above. Automatic 3D gradient echo shimming was initially used to adjust B0 homogeneity in the voxel and further manually shimmed to water linewidths of 10–13 Hz (~0.03–0.04 ppm). The water signal was suppressed using variable power radiofrequency pulses with optimized relaxation delays (VAPOR) to obtain B1 and T1 insensitivity. A PRESS sequence (TE = 19.76 ms) was used for the pre-localization. Data were collected by averaging 512 excitations (frequency corrected 32 × 16 scan blocks) with TR of 4 s, number of complex pairs 3,000, and spectral width 3 kHz (10 ppm). In addition, reference spectra without water suppression (16 averages) were collected from the identical voxel using the same acquisition parameters. Peak areas for the major metabolites, N-acetylaspartate, choline, taurine, inositol, glutamate, glutamine, creatine and phosphocreatine, were analyzed using LCModel 8 (Stephen Provencher Inc., Oakville, ON, Canada), and the results are given relative to water content in the tissue.

### 2.5. Biochemical measurements

#### 2.5.1. Brain tissue preparation and RNA extraction

In 1–3 days after ^1^H-MRS, the rats from the MWM cohort were deeply anesthetized with pentobarbital and transcardially perfused with heparinized saline. In five aged rats treated with vehicle, seven aged rats treated with VH-04, and nine young adult control rats treated with vehicle, the left hemisphere was dissected on ice to obtain the hippocampus, fresh-frozen on dry ice and stored at −80°C. Purification of total RNA was performed by RNeasy Protect Mini Kit (Qiagen). For the analysis of synaptophysin (*Syp*) mRNA expression levels, the hemispheres were thawed on ice, and sample portions weighing 20–30 mg were cut. These samples were placed into 1.5 mL tubes and homogenized in 600 μL of buffer RLT (Qiagen, Hilden, Germany) by a Kontes Pellet Pestle homogenizer until the lysate was uniformly homogenous. The lysate was centrifuged for 3 min at 10,000 rpm/8,000 *g*, and the supernatant was removed by pipetting into a new 1.5 mL tube. One volume of 70% ethanol was added, and 700 μL of the resulting sample, including any precipitate, was transferred to an RNeasy spin column (cat. #74126, Qiagen) placed in a 2 mL collection tube and centrifuged (15 s, 10,000 rpm/8,000 *g*). Next, 700 μL of buffer RW1 (Qiagen) was added to the column, which was centrifuged again (15 s, 10,000 rpm/8,000 *g*) to wash the column membrane. To eliminate contamination with genomic DNA, the membranes were treated with DNAse I. After treatment with DNase I incubation mix, 350 μL of buffer RW1 was added to the RNeasy spin column and centrifuged (15 s, 10,000 rpm/8,000 *g*). After that, the spin column membrane was washed twice by adding 500 μL of buffer RPE and centrifuged (15 s, 10,000 rpm/8,000 *g*). RNA from the membrane was eluted with 30 μL of RNase-free water (1 min, 10,000 rpm/8,000 *g*) and stored at −20°C. The RNA concentration and quality were determined by spectrophotometry (NanoDrop 1,000, Thermo Fisher Scientific, Waltham, MA, United States).

#### 2.5.2. cDNA synthesis

The SuperScript III First-Strand Synthesis System (Invitrogen, Carlsbad, CA, United States) was used to synthesize first-strand cDNA from total RNA. Total RNA (500 ng) was resuspended in molecular grade water to a final volume of 11 μL and 1 μL of random hexamers, and 1 μL of the dNTP mix was added. Samples were incubated at 65°C for 5 min, and the tubes were placed on ice for 2 min. Then, 2 μL of 10× First Strand Buffer, 1 μL of 0.1 M dithiothreitol, 1 μL of the Superscript enzyme, and 3 μL water was added. After that, the samples were incubated at 25°C for 5 min, at 50°C for 50 min, at 70°C for 15 min and left at +4°C. After cDNA synthesis, the samples were stored at −20°C.

#### 2.5.3. RT-qPCR

The reaction mix contained 12.5 μL of TaqMan^®^ Gene Expression Master Mix, 2 μL of *Syp* Assay on Demand, 2 μL of template DNA sample and 8.5 μL of RNAse/DNAse-free water. The combined reagent mix was added to a well of a 96-well plate, and an ABI 7500 Real Time PCR Machine (Applied Biosystems) and 7,500 Software v.2.0.5 were used for the quantitative analysis of *Syp* mRNA expression in the samples. The expression level of the synaptophysin mRNA was normalized by *Gapdh* mRNA levels.

### 2.6. Mouse primary hippocampal neurons

#### 2.6.1. Isolation of E18 hippocampal neurons

For primary cell culture experiments, C57Bl/6 J mice at embryonic day 18 (E18) were used. Briefly, pregnant mice were sacrificed according to the general guidelines for animal experimentation by cervical dislocation. E18 embryos were extracted from the uterus and sacrificed by decapitation. The whole brains were removed from the skull and transferred to a culture dish containing ice cold 1× Hank’s Balanced Salt Solution (HBSS) without Ca^2+^, Mg^2+^ and phenol red (Gibco #4175095). The next steps were carried out on ice. The hemispheres were separated, meninges were removed, and the hippocampi were dissected from the remaining brain tissue using fine forceps under a Zeiss Stemi 2000-C stereomicroscope. Isolated hippocampi were collected in a sterile 15 mL tube containing ice-cold 1× HBSS. Before dissociation, hippocampi were washed thrice with 10 mL ice cold 1× HBSS. Then, the excess of 1× HBSS was removed to leave 2 mL with isolated hippocampi, and 0.2 mL of 2.5% trypsin was added and incubated for 20 min in a 37°C water bath. Subsequently, the hippocampi were washed thrice gently with 10 mL 1× HBSS, and the excess of 1× HBSS was removed to leave 2 mL with isolated hippocampi. Then 65 U/mL DNAse I was added and hippocampi were mechanically dissociated by pipetting using a 1,000 μL tip until the suspension became homogenous. The cell suspension was transferred to a 50 mL tube through a 100 μm cell strainer. Dulbecco’s Modified Eagle’s Medium (DMEM) (Gibco #41966) supplemented with 10% fetal calf serum, 2 mM L-glutamine, 100 IU penicillin and 0.1 mg/mL streptomycin was added, and the cells were counted using a Neubauer cell counting chamber. The concentration of cells in the suspension was adjusted to 8 × 10^4^ cells per mL. The cells were seeded in 24-well cell culture plates containing poly-L-lysine-coated 13 mm round cover slips. The total cell density was 2.5 × 10^4^ cells per 1 cm^2^.

#### 2.6.2. Cell culture and analysis of neuron morphology

Primary hippocampal neurons were maintained at 37°C in an atmosphere of 95% air and 5% CO_2_. The medium was changed in 3–18 h after plating. The DMEM adhesion medium was removed and 600 μL of the Neurobasal culture medium supplemented with 10% B27, 2 mM L-glutamine, 100 U penicillin, and 0.1 mg/mL streptomycin was added. The effect of VH-04 on the morphology of primary hippocampal neurons (8 × 10^4^ cells/well) was tested after incubation with the drug for 3, 5 or 7 days at dilutions (medium:VH-04 or vehicle) 1:3, 1:4 (for 3 days), and 3:1 (5 and 7 days). Saline was added as vehicle to the wells with control neurons. In the end of the incubation time, primary hippocampal neurons were fixed in 4% paraformaldehyde/4% sucrose, washed in DPBS without Ca^2+^ and Mg^2+^ and permeabilized in a 0.2% Triton-X solution in phosphate buffered saline for 10 min. Then, the neurons were incubated in 1× Immunoblock (Carl Roth, Karlsruhe, Germany) for 1 h at room temperature and subsequently with an anti-MAP2 antibody (GeneTex, Irvine, CA, United States, #GTX25392) (diluted 1:10,000 in 1× Immunoblock) at 4°C overnight. The next day after three washes with phosphate buffered saline, the cells were incubated with a goat anti-chicken Alexa Fluor 488 (Invitrogen #A11039) secondary antibody (Molecular Probes, Invitrogen, United States) (diluted 1:750 in Immunoblock). The round cover slips with primary hippocampal neurons were covered with Mowiol (mounting agent) and analyzed with an Axiovert 200 fluorescence microscope using AxioVision software rel 4.8 (Zeiss, Oberkochen, Germany).

Neurite length was analyzed with ImageJ software using NeuronJ plugin. For every incubation time and dilution, between 19 and 70 cells from 3–4 different wells were analyzed (3 days, 1:3: *n* = 20 for both VH-04 and vehicle treatments; 3 days, 1:4: *n* = 20 for vehicle and *n* = 19 for VH-04; 5 days, 3:1, single treatment: *n* = 40 for both VH-04 and vehicle; 5 days q.d., 3:1: *n* = 39 for both VH-04 and vehicle; 7 days, 3:1: *n* = 70 for both VH-04 and vehicle). Prepared microscopy slides were imaged with the appropriate filters using a Zeiss Axiovert 200 inverted microscope with a 100× oil immersion objective and every neurite/dendrite was analyzed by NeuronJ software by drawing the neurite length.

### 2.7. Statistical analysis

Data in the text are presented as the mean ± standard deviation. Cognitive test outputs in multiple treatment groups were assessed using one- or two-way analysis of variance (ANOVA), depending on the number of factors affecting the parameters. *Post hoc* Dunnett’s or Holm–Šídák multiple comparisons tests followed ANOVA if *F* value indicated a significant effect of the factor. The Dunnett’s test was used when effects of multiple treatments were compared to a value in a single reference group (vehicle- or scopolamine-treated, depending on the test). The Holm-Šídák multiple comparisons test was performed if all treatment groups were compared between themselves. Two-way repeated measures ANOVA was used to analyze the effects of VH-04 on the time course of spatial learning parameters in the MWM task. Ordinary two-way ANOVA was used to assess the outcomes social transmission of food preference test with food and treatment as independent factors. Pairwise comparisons of neurite lengths between treatment groups were performed using the unpaired Student’s or Welch’s *t*-test, depending on the equality of variances. Brown-Forsythe ANOVA was used to analyze *Syp* mRNA levels. Effects were considered statistically significant if *p* < 0.05. All statistical analyses were performed using GraphPad Prism v. 9.0 (GraphPad Software, San Diego, CA, United States).

## 3. Results

### 3.1. Effects of VH-04 on rodent cognitive behavior

#### 3.1.1. VH-04 improves visual recognition memory

**Figure 2:**
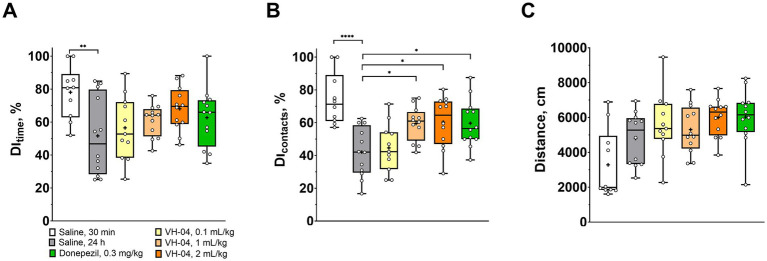
VH-04 positively modulates visual recognition memory. **(A,B)** Discrimination indices (DIs) based on the time **(A)** and number of contacts **(B)** with the novel object during the retention trial in 30 min or 24 h after the initial acquisition trial in rats that received saline, VH-04, or donepezil. **(C)** Distances ran by rats during the retention trial. Data are presented as box-whisker plots (middle line: median; box: 25th and 75th percentiles; cross: mean value; whiskers: smallest and largest values; *N* = 10–12). Statistical significance of differences was determined by one-way ANOVA followed by the Dunnett’s multiple comparisons test (vs. “Saline, 24 h) and is illustrated as follows: *p < 0.05; **p < 0.01; ****p < 0.0001 (see Supplementary Table 1 for the full breakdown of statistical results).

The NOR test is based on the innate tendency of rodents to pay more attention to a novel object than to a familiar one in the same experimental setting. It assesses the capacity for recognition memory by comparing the duration and number of physical contacts of the animal with these two types of objects. In our experiments, control saline-treated rats demonstrated a high level of discrimination and spent over 78 ± 16% of time investigating a novel object when it was presented in 30 min after the acquisition trial (Figure 2A). Expectedly, in control rats, for which the delay between the acquisition and retention trials was extended to 24 h, discrimination index based on the time of interaction (DI_time_) decreased to 52 ± 24% (*p* = 0.0034, Dunnett’s test). In animals that received intraperitoneal administrations of VH-04, DI_time_ during the 24 h retention trial was dose-dependently higher than in saline-treated rats, but the differences did not reach statistical significance. Similar results were obtained when discrimination was assessed from the number of contacts animals made with the novel object (Figure 2B). Treatment had an overall significant effect on DI_contacts_ [*F*_(5, 63)_ = 7.29; *p* < 0.0001], and statistically higher DI_contacts_ values were observed in rats treated with 1 or 2 mL/kg VH-04 than in saline-treated rats (*p* = 0.0199 and 0.0133, respectively; Dunnett’s test; Figure 2B). The cholinesterase inhibitor donepezil, which is one of the few drugs approved to treat dementia symptoms in Alzheimer’s disease and several other neurodegenerative disorders (Kim et al., 2017; Meng et al., 2019), also increased the DI_contacts_ value compared to that observed in saline-treated rats (*p* = 0.0184, Dunnett’s test; Figure 2B). It is unlikely that VH-04 effect on the visual recognition memory was caused by any changes in the locomotion, as rats that received any dose of the drug and vehicle-treated mice ran similar distances during the 24 h retention trial (Figure 2C).

#### 3.1.2. VH-04 improves spatial working memory after scopolamine-induced impairment

**Figure 3:**
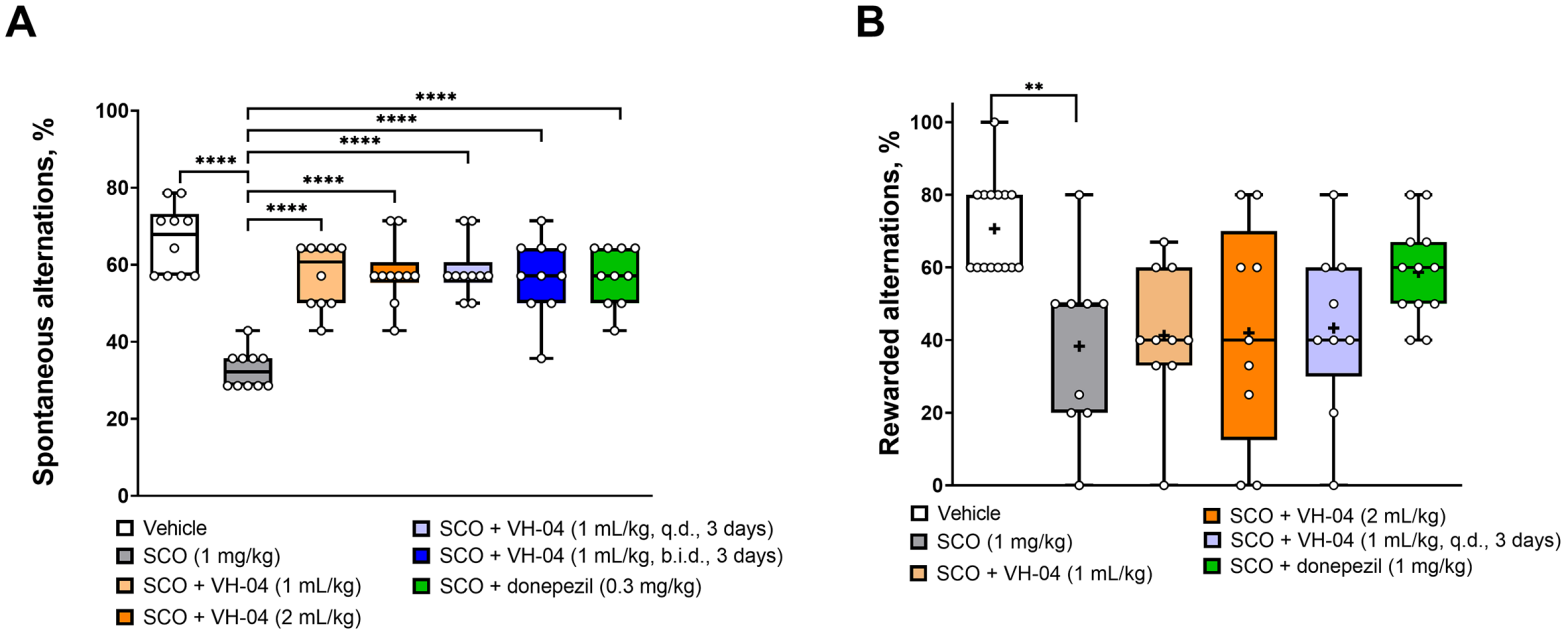
Modulation of the spatial working memory by VH-04. Drug effects were determined in the T-maze spontaneous **(A)** and rewarded **(B)** alternation tests in CD1 mice and Wistar rats, respectively. Data are presented as box-whisker plots (middle line: median; box: 25th and 75th percentiles; cross: mean value; whiskers: smallest and largest values). For experiments in **(A)**, *N* = 10 mice per each group. For experiments in **(B)**, *N* = 9–15 rats in each group. Statistical significance of differences was determined by one-way ANOVA followed by the Dunnett’s multiple comparisons test [vs. “SCO (1 mg/kg)”]: **p < 0.01; ****p < 0.0001 (see Supplementary Table 2 for the full breakdown of statistical results). b.i.d., twice a day; q.d., daily.

To examine how VH-04 modulates the spatial working memory, we performed experiments on T-maze spontaneous alternation in mice and rewarded alternation in rats. T-maze tests exploit the natural tendency of rodents to explore unfamiliar areas, which in the context of a maze means that animals more likely visit the previously unattended arm of the T-maze than the previously visited arm. The ability to differentiate between previously visited and unvisited arms is thought to correlate with the capacity for spatial working memory, and it is assessed by calculating the fraction of alternations (i.e., visits to the previously unvisited arm) out of all trials in the experiment. In the rewarded alternation test, the tendency to select the previously unvisited arm is further stimulated by a small nutritional reward. In our experiments, we were interested in whether VH-04 may help restoring spatial memory deficit caused by the injection of the nonselective muscarinic antagonist scopolamine, a treatment, which is commonly used to model central cholinergic deficits in aged individuals or patients with Alzheimer’s disease (Buccafusco, 2009). In the T-maze spontaneous alternation test (Figure 3A), mice that received acute intraperitoneal injection of 1 mg/kg scopolamine demonstrated a two-fold lower fraction of spontaneous alternations compared to that in control animals (33 ± 5% vs. 66 ± 9%, *p* < 0.0001, Dunnett’s test). In mice that received single or multiple administrations of VH-04, or a single injection of donepezil before scopolamine administration, the fractions of spontaneous alternations were significantly greater than that in mice injected with scopolamine only (*p* < 0.0001 in all cases; Figure 3A).

Administration of scopolamine to rats significantly decreased the fraction of the nutritionally rewarded alternations from 71 ± 13% to 38 ± 24% (*p* = 0.0017, Dunnett’s test; Figure 3B). However, in contrast to its memory-improving effect in the spontaneous alternation test, pretreatment with VH-04 failed to significantly increase the percentage of rewarded alternations suppressed by scopolamine. In the same setting, rats that received injections of both scopolamine and donepezil had a numerically higher performance (59 ± 13% of rewarded alternations) than animals that were injected with scopolamine only, but the effect of the cholinesterase inhibitor did not achieve the set level of statistical significance (*p* = 0.099, Dunnett’s test).

#### 3.1.3. VH-04 improves retention of spatial orientation memory in old rats in the MWM test

**Figure 4:**
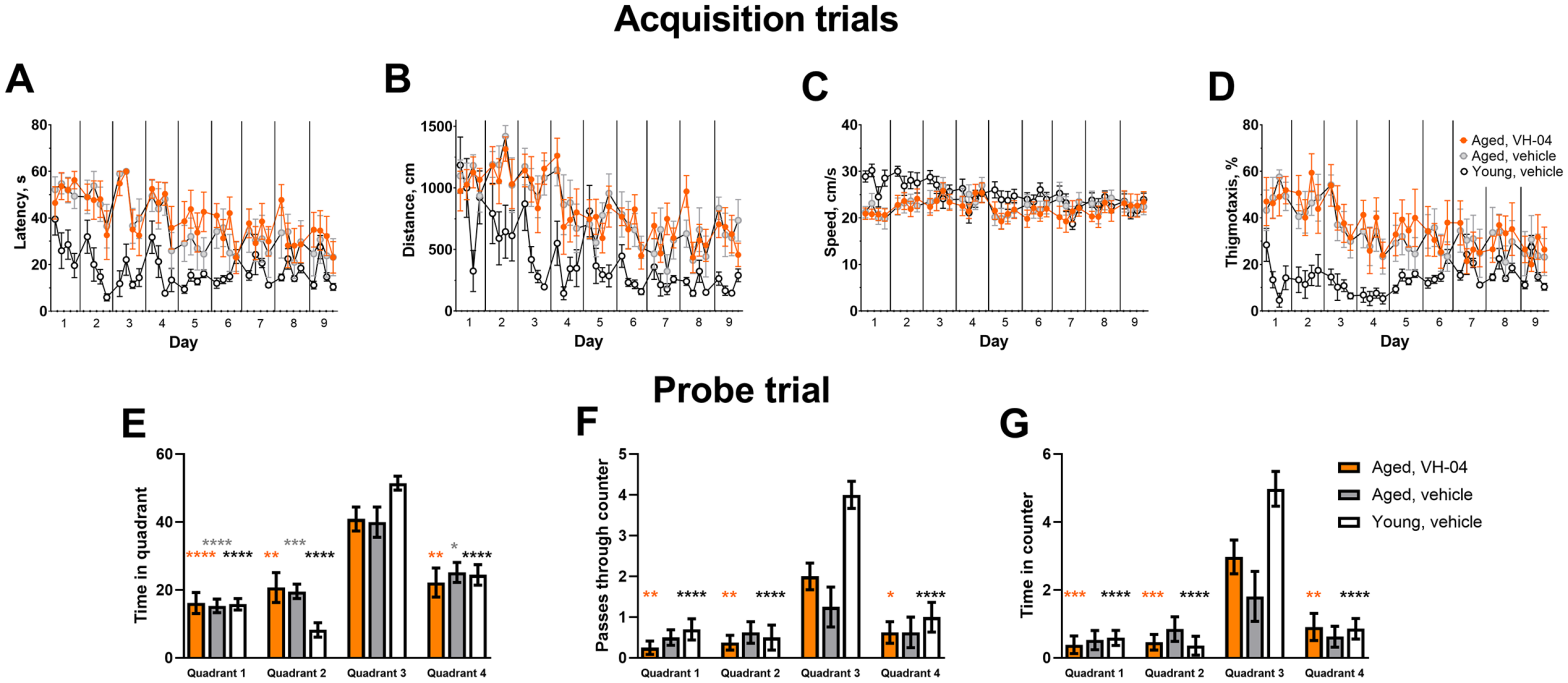
Effect of VH-04 administration on spatial orientation learning and memory retention in the Morris water maze. **(A–D)** Latency **(A)** and distance **(B)** to reach the platform, speed **(C)**, and the extent of thigmotaxis **(D)** during acquisition trials in vehicle-treated young rats (3 months), and in vehicle- or VH-04-treated aged rats (25 months) trained in the Morris water maze. Data are presented as the mean ± S.E.M. (*N* = 8–10 rats in each group). Two-way repeated measures ANOVA and subsequent Holm-Šídák multiple comparisons did not reveal significant differences in any of the four parameters between aged rats treated with vehicle or VH-04 at any of the acquisition trials. **(E–G)** Time spent in different quadrants **(E)**, number of passes through the platform counter **(F)**, and time spent within the platform counter area **(G)** during the probe trial. Data are presented as the mean ± S.E.M. (*N* = 8–10 rats in each group). Data in **(E–G)** were analyzed by two-way ANOVA followed by the Holm-Šídák multiple comparisons test to compare values in each quadrant within the same experimental group. Statistical significance of differences from the values in target quadrant 3 is illustrated as follows: *p < 0.05; **p < 0.01; ***p < 0.001; ****p < 0.0001 (see Supplementary Table 3 for the full breakdown of statistical results).

To assess how VH-04 influences spatial orientation learning and memory, which is often impaired with age, we carried out experiments in young (3 months) and old (25 months) Sprague Dawley rats that learned to find the position of the underwater platform in the MWM using visual cues around the maze. If the rat finds the underwater platform with increasingly faster latency and shorter distance during the successive acquisition trials, this is considered as evidence of spatial orientation learning. After the animal has learned to find the platform, the latter is removed during a probe trial, and spatial orientation memory for the platform location is inferred from the time spent in the right part of the MWM and number of crossings of the area previously occupied by the platform. In our experiments, during acquisition sessions (Figures 4A,B), both group and trial number had significant effects on the latency and distance to reach the platform, reflecting expectedly superior performance of young rats and gradual learning of the task by all animals (Supplementary Table 3). However, Holm-Šídák multiple comparison tests revealed no significant differences at respective acquisition trials between aged rats treated with vehicle or VH-04 in any of the parameters (Supplementary Table 3), indicating that the drug did not appreciably affect spatial orientation learning (Figures 4A,B) or locomotor behavior (Figures 4C,D) in the acquisition phase. During the probe trial, when the platform was removed from the pool, all groups showed a clear preference for the target quadrant 3, where they spent most of the time (Figure 4E). However, despite swimming predominantly in the correct quadrant, vehicle-treated aged rats did not cross or stayed within the boundaries of the platform counter area significantly more than in counter areas of other quadrants (Figures 4F,G), reflecting impaired retention of the spatial orientation memory (*p* > 0.05 in all comparisons). At the same time, VH-04-treated aged rats crossed the platform counter area significantly more frequently and spent more time in the platform counter area than in equivalent counter areas of other quadrants (*p* < 0.05 for all comparisons), which demonstrated improved retention of spatial orientation memory about platform location (Figures 4F,G).

#### 3.1.4. VH-04 does not ameliorate scopolamine-induced impairment of fear-aggravated memory

To investigate modulation of the association memory, we examined the effects of VH-04 in the fear-aggravated passive avoidance test and contextual/cued fear conditioning.

##### 3.1.4.1. Passive avoidance test

**Figure 5:**
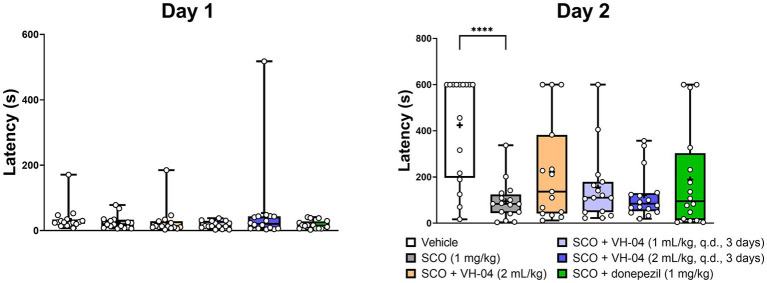
Administration of VH-04 does not improve passive avoidance performance impaired by scopolamine. VH-04 was given either simultaneously with scopolamine or for 3 days q.d. before the treatment with scopolamine. Latencies to enter the dark chamber on the training day (Day 1) and next day (Day 2) for different experimental groups of rats are illustrated. Data are presented as box-whisker plots (middle line: median; box: 25th and 75th percentiles; cross: mean value; whiskers: smallest and largest values; *N* = 15 or 16 rats in each group). Statistical significance of differences was determined by one-way ANOVA followed by the Dunnett’s multiple comparison test (vs. “SCO (1 mg/kg)”): ****p < 0.0001 (see Supplementary Table 4 for the full breakdown of statistical results). q.d., daily.

In the passive avoidance test, animals experienced a foot shock following their visit to a dark chamber on training day 1, and they were tested for the latency to enter this chamber in 24 h. The reluctance to enter the dark chamber for longer periods on day 2 gauges association memory of the foot shock given on the previous day. In our experiments, the majority of vehicle-treated rats refrained from entering the dark chamber for over 400 s on day 2, despite on day 1, this and other experimental groups visited the dark chamber within 18–54 s on average (Figure 5). The rats that received 1 mg/kg scopolamine 30 min before training on day 1 showed impaired association memory on day 2 and visited the dark chamber after 95 ± 84 s, which was significantly faster compared to the latency seen in vehicle-treated rats (*p* < 0.0001, Dunnett’s multiple comparisons test). Pretreatment with VH-04 at a range of doses or donepezil failed to significantly increase the latencies to enter the dark chamber in scopolamine-treated rats in comparison to the values observed in animals that received scopolamine only (*p* > 0.05 in all cases; day 2; Figure 5).

##### 3.1.4.2. Contextual/cued fear conditioning test

**Figure 6:**
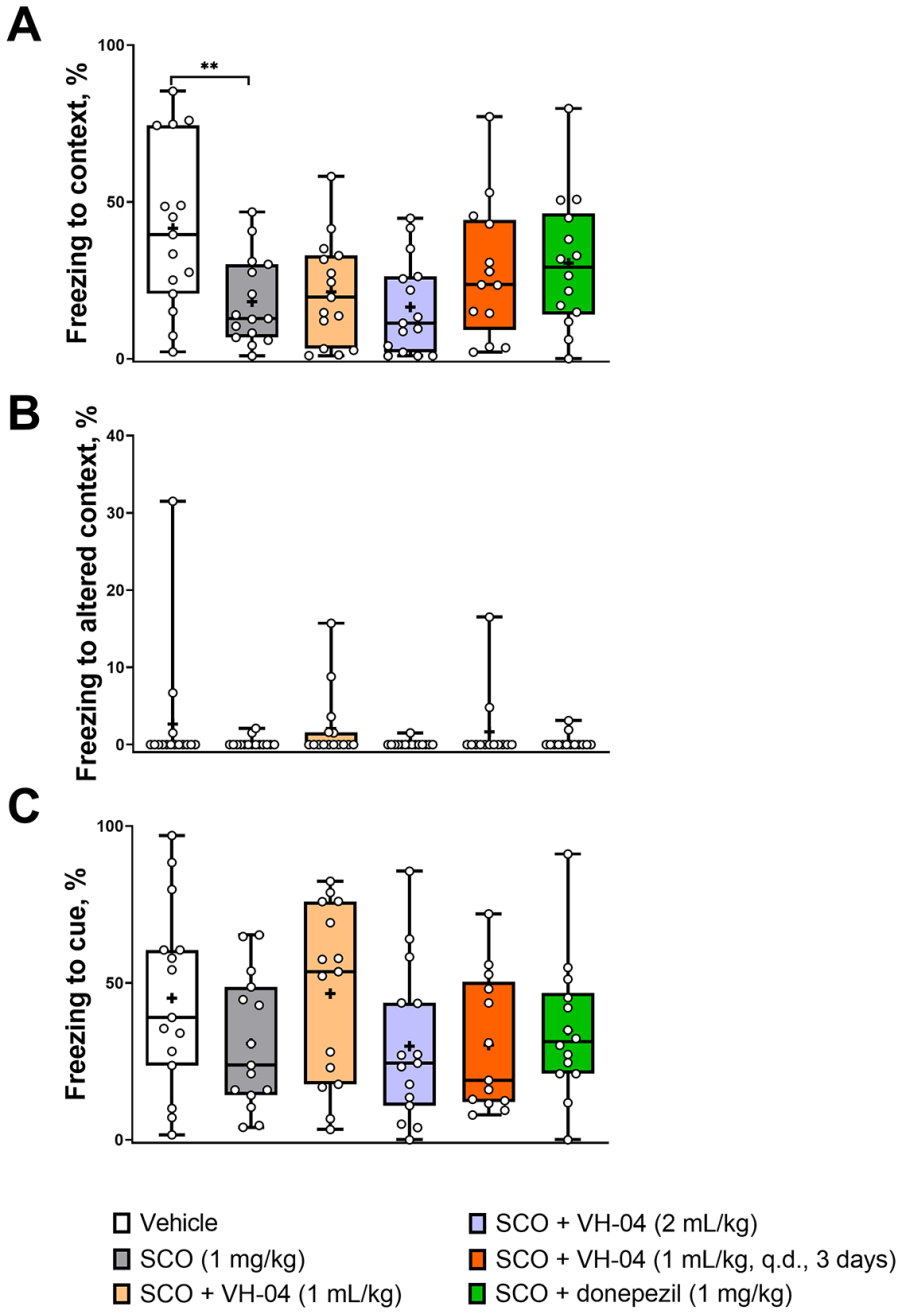
VH-04 administration does not ameliorate the amnesic effect of scopolamine in contextual/cued fear conditioning. Fractions of time spent freezing to context **(A)**, altered context **(B)**, and cue in altered context **(C)** are shown. Data are presented as box-whisker plots (middle line: median; box: 25th and 75th percentiles; cross: mean value; whiskers: smallest and largest values; *N* = 13–15 mice in each group). Statistical significance of differences was determined by one-way ANOVA followed by the Dunnett’s multiple comparisons test (vs. “SCO (1 mg/kg)”): **p < 0.01 (see Supplementary Table 5 for the full breakdown of statistical results). q.d., daily.

During the contextual/cued fear conditioning test, rodents are placed into an operant chamber, where they receive a series of aversive electric shocks (unconditional stimulus) and learn to associate them with the experimental setting (context) and a neutral auditory stimulus (conditional stimulus/cue). The capacity for the fear memory is assessed from the tendency of the animal to display lack of mobility (“freezing”) as the conditional response in anticipation of harm, when it is exposed to the same context (same operant chamber) or cue (same sound) after a delay following the conditioning episode. In our experiments, C57Bl/6 J mice received an aversive foot shock as an unconditional stimulus, which was paired with a neutral auditory conditional stimulus on day 1 and were tested for freezing behavior again in 20 h after conditioning. The mice were initially placed in the same chamber as during conditioning on the previous day to assess memory for context. Then, they were placed in a different, novel chamber (altered context), where their freezing behavior was monitored first without and then with the conditional auditory stimulus to assess memory for cue. Control, vehicle-treated mice spent more than 40% of time freezing on day 2 when they were placed in the same context or heard the cue sound in an altered context (Figures 6A,C), whereas in the absence of the conditional stimulus, freezing in the altered context was minimal (Figure 6B). Mice that were injected with 1 mg/kg scopolamine 30 min before the training on day 1, spent less time freezing on day 2 during the exposure to the same context (18 ± 14%) or cue (31 ± 21%) than vehicle-treated mice (Figures 6A,C), which indicated impairment of cognitive functions by scopolamine treatment. The effect of scopolamine, however, was statistically significant only for the contextual memory (*p* = 0.0074, Dunnett’s multiple comparisons test). Neither pretreatment with VH-04 at different doses nor co-injection of donepezil significantly increased the amount of context-dependent freezing in scopolamine-treated mice compared to the level of freezing seen in animals that received only scopolamine (*p* > 0.05 for all comparisons, Dunnett’s multiple comparisons test). As for the response to cue, the treatment factor did not significantly affect freezing to cue [*F*_(5, 81)_ = 1.426, *p* = 0.2239].

#### 3.1.5. VH-04 improves olfactory memory after scopolamine-induced impairment

**Figure 7:**
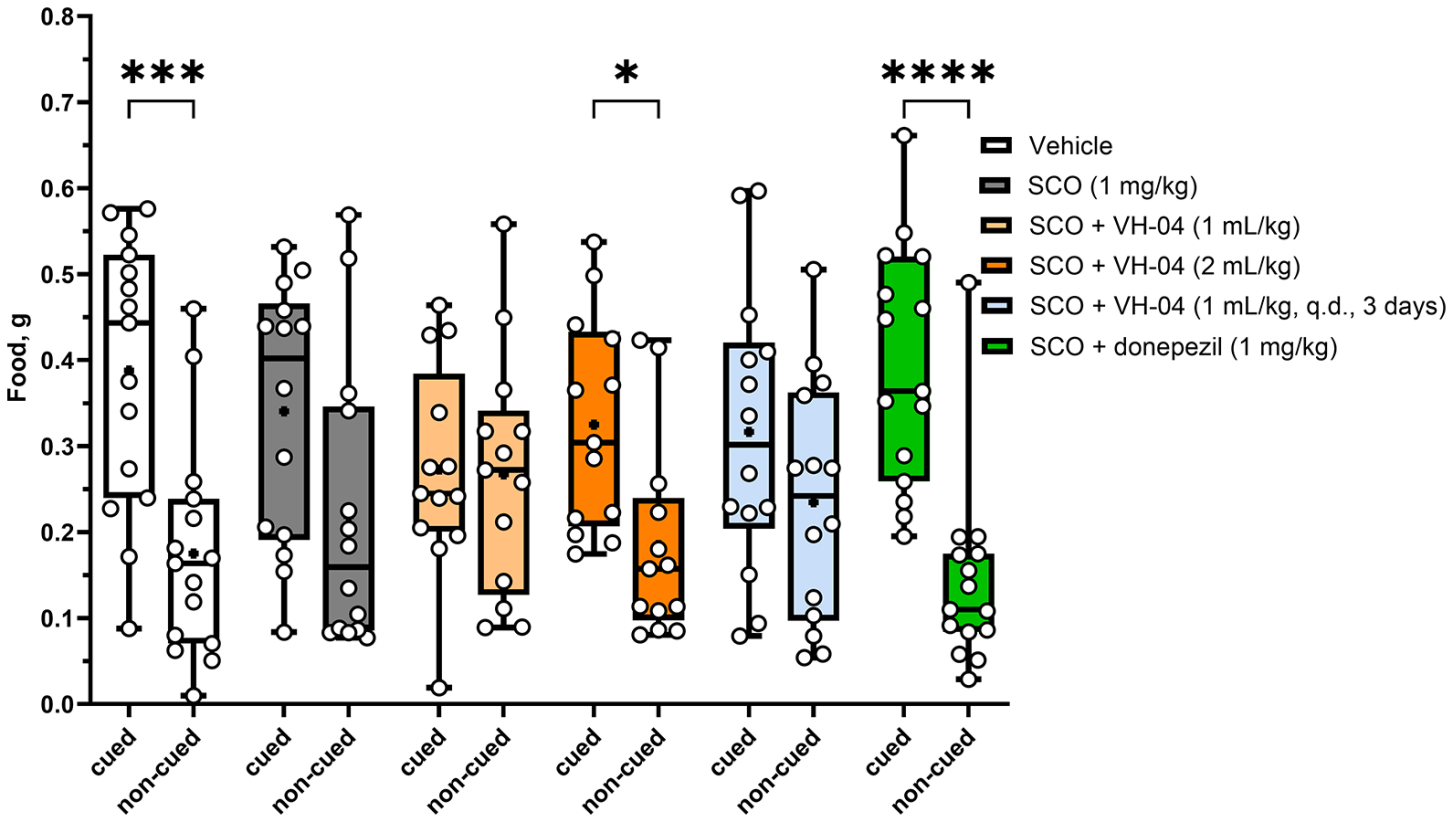
Restoration of the preference for cued food impaired by scopolamine following treatment with VH-04 or donepezil in the social transmission of food preference test. Absolute amounts of cued and non-cued food consumed by “observer” mice are illustrated. Data are presented as box-whisker plots (middle line: median; box: 25th and 75th percentiles; cross: mean value; whiskers: smallest and largest values; *N* = 13–15 mice in each group). Ordinary two-way ANOVA with food and treatment type as factors showed a significant effect of factor interaction [*F*_(5, 156)_ = 2.818, *p* = 0.0182]. Asterisks indicate groups in which the difference in the amount of cued and non-cued food was significant according to the Holm-Šídák multiple comparisons test: *p < 0.05; ***p < 0.001; ****p < 0.0001 (see Supplementary Table 6 for the full breakdown of statistical results). q.d., daily.

The STFP test was used to establish whether VH-04 modulates olfactory memory. In this test, “observer” mice interact with “demonstrator” mice that had consumed a novel food shortly before the encounter. When an “observer” mouse, in turn, is presented with a choice of eating either the novel food eaten by the “demonstrator” mouse or another new food, it usually prefers the former based on the memory for olfactory cues. This phenomenon relies on the natural assumption that it is safer to eat that novel food, the scent of which had been already presented in the breath of a healthy group member. The hippocampal memory, crucial for successful performance in the STFP test, could be compared to the declarative memory in humans. In our experiments, following the contact with corresponding “demonstrator” mice, which were former cage mates, the vehicle-treated “observer” mice consumed significantly more cued food previously sampled by the “demonstrator” compared to the amount of eaten non-cued food (*p* = 0.0002; Holm-Šídák’s multiple comparisons test; Figure 7). Mice administered with scopolamine also consumed nominally more cued food, but the difference between the amounts of consumed cued and non-cued food was not significant (*p* = 0.065). We attempted to restore the level of preference for the cued food by three different treatments with VH-04 and observed that the difference between the amounts of cued and non-cued food became significant after administration of VH-04 at 2 mL/kg (*p* = 0.0452; Figure 7). In the same setting, 1 mg/kg donepezil also restored significant preference for cued food in scopolamine-treated animals (*p* < 0.0001; Figure 7).

In summary, on a behavioral level, VH-04 improved visual recognition memory after a single administration in the NOR test and spatial orientation memory in aged animals after repeated administrations in the MWM probe trial. VH-04 also ameliorated impairments in spatial working memory (T maze spontaneous alternations test) and olfactory memory (STFP) induced by the muscarinic antagonist scopolamine (Table 1).

**Table 1:** Summary of VH-04’s effects on cognitive performance.

Cognitive parameter	Behavioral test	Species	Result
Visual recognition memory	Novel object recognition	Rats	Cognitive improvement
Scopolamine-induced impairment of spatial working memory	T-maze spontaneous alternation	Mice	Cognitive improvement
Scopolamine-induced impairment of spatial working memory	T-maze rewarded alternation	Rats	No effect
Scopolamine-induced impairment of fear-aggravated memory	Passive avoidance	Rats	No effect
Scopolamine-induced impairment of fear-aggravated memory	Contextual/cued fear conditioning	Mice	No effect
Scopolamine-induced impairment of olfactory memory	Social transmission of food preference	Mice	Cognitive improvement
Spatial orientation learning and memory in aged animals	Morris water maze	Rats	No effect (spatial orientation learning) Cognitive improvement (spatial orientation memory)

### 3.2. Effects of VH-04 on the molecular and cellular parameters

#### 3.2.1. VH-04 does not alter hippocampal metabolic profile in aged rats

**Figure 8:**
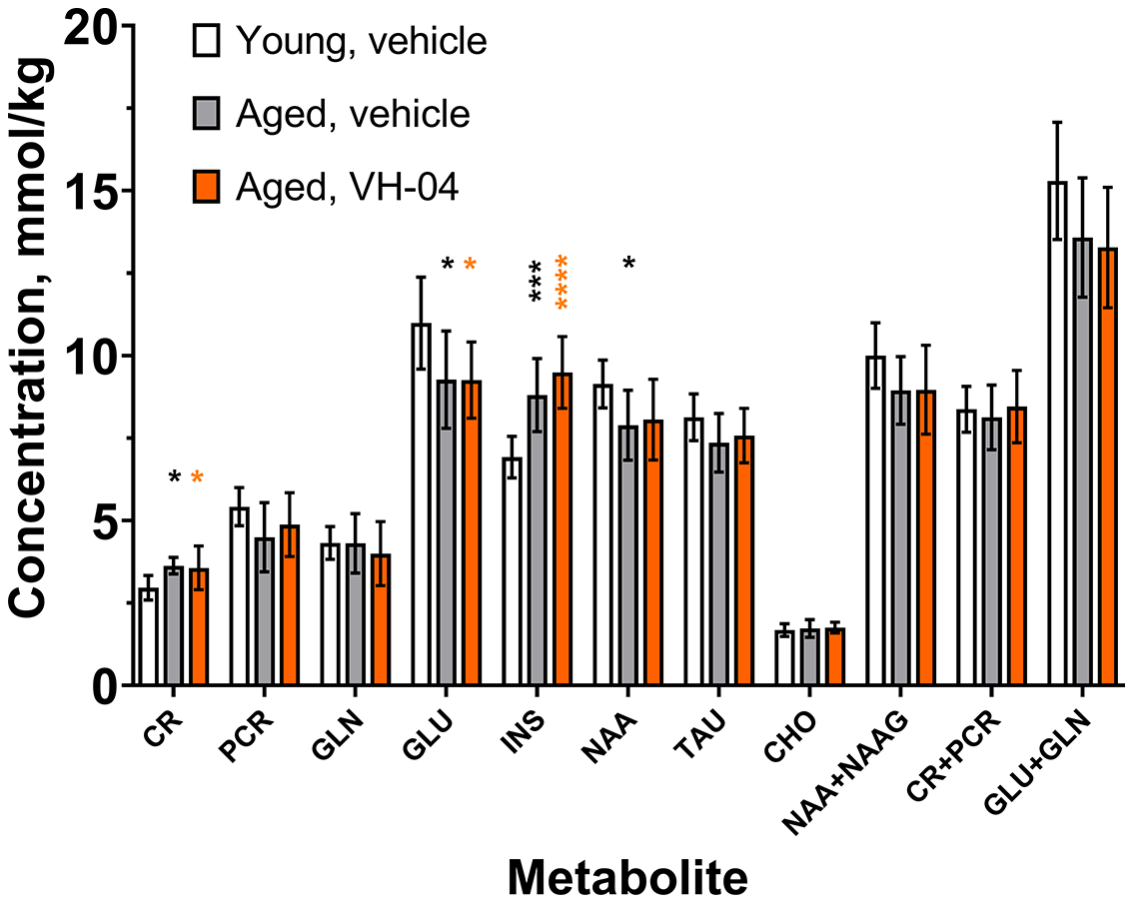
Age-related changes in the concentrations of various metabolites in the hippocampal region of rats are not restored by VH-04 administration. Measurements were performed in animals used in the Morris water maze test (Figure 3) in 1–4 days following the probe trial. Data are presented as the mean ± S.D. (*N* = 8 or 10 mice in each group). Data for each metabolite were analyzed by one-way ANOVA followed by the Holm-Šídák multiple comparisons test. Statistical significance of differences from the values in vehicle-treated young rats is illustrated as follows: *p < 0.05; ***p < 0.001; ****p < 0.0001 (see Supplementary Table 7 for the full breakdown of statistical results). CHO, choline; CR, creatine; GLN, glutamine; GLU, glutamate; INS, *myo*-inositol; NAA, *N*-acetyl-aspartate; NAAG, *N*-acetyl-aspartyl-glutamate; PCR, phosphocreatine; TAU, taurine.

^1^H-MRS was used to determine concentrations of various metabolites in the hippocampal region of the young and aged rats that were used in experiments with Morris water maze (Figure 8). Group factor significantly affected the levels of creatine [*F*_(2, 23)_ = 6.046, *p* = 0.0078], glutamate [*F*_(2, 23)_ = 4.975, *p* = 0.016], inositol [*F*_(2, 23)_ = 18.24, *p* < 0.0001], and *N*-acetylaspartate [*F*_(2, 23)_ = 4.227, *p* = 0.0273]. *Post hoc* Holm-Šídák multiple comparisons showed that for all these four metabolites, the difference between the levels in vehicle-treated young and aged rats was statistically significant. At the same time, treatment of aged rats with VH-04 did not significantly change metabolite levels compared to those in vehicle-treated aged rats. We noted however, that in aged rats treated with VH-04, the level of *N*-acetylaspartate was not significantly different from that in vehicle-treated young rats (Figure 8).

#### 3.2.2. VH-04 potentiates neurite outgrowth in mouse primary hippocampal neurons

**Figure 9:**
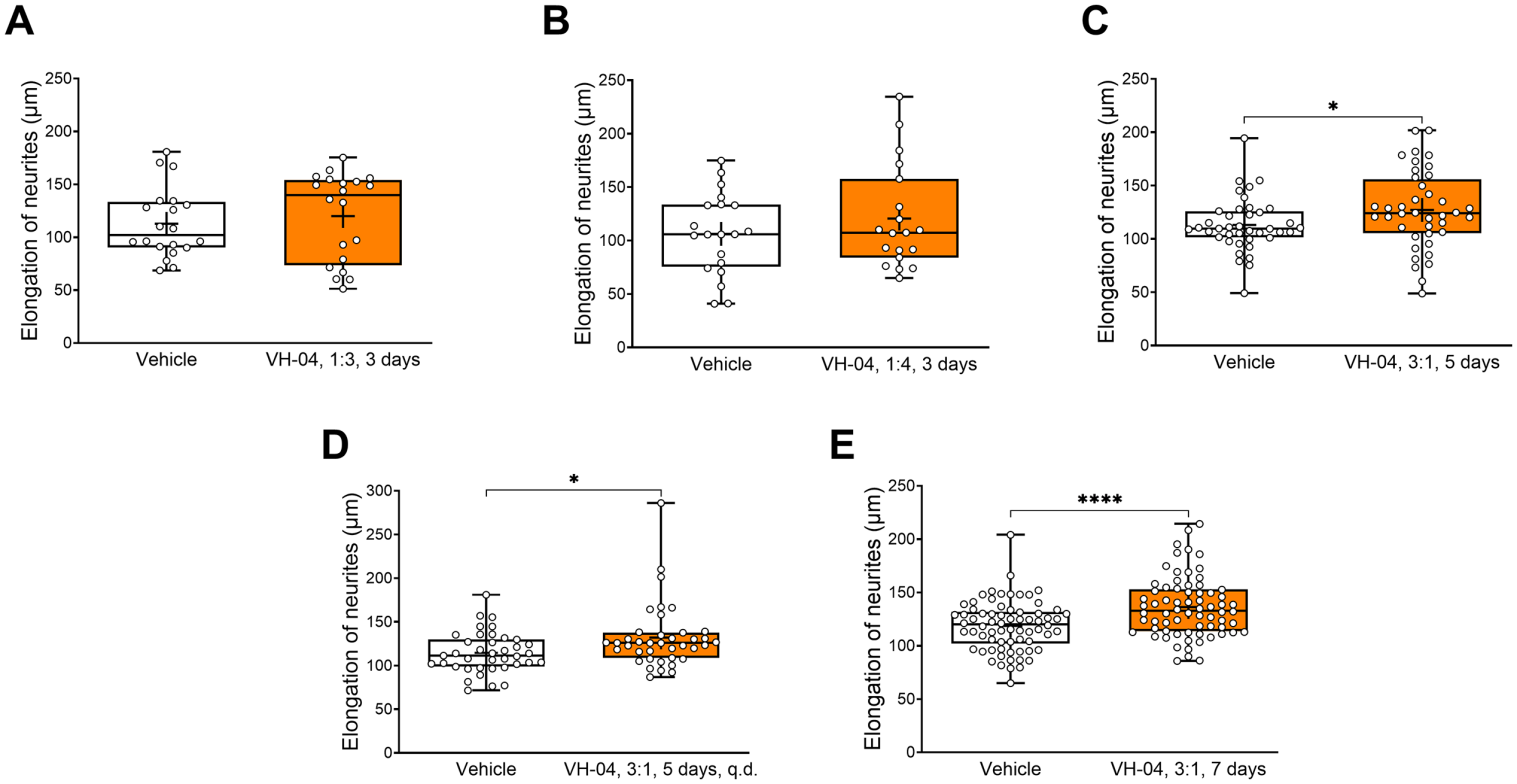
Positive effect of incubation with VH-04 on neurite length in mouse primary hippocampal neurons. Data are presented as box-whisker plots (middle line: median; box: 25th and 75th percentiles; cross: mean value; whiskers: smallest and largest values; *N* = 19–70 neurites in each group). Statistical significance of differences was determined by the unpaired Student’s or Welch’s *t*-test, depending on the equality of variances: *p < 0.05; ****p < 0.0001 (see Supplementary Table 8 for the full breakdown of statistical results).

Incubation of primary hippocampal neurons obtained from embryonic day 18 C57Bl/6 J mouse embryos with VH-04 at various concentrations (1:4, 1:3, and 3:1) showed that the drug positively affected neurite growth at all tested concentrations and regimens (Figure 9). However, the increases in neurite length were significantly higher than in vehicle-treated neurons only in cells that were treated with VH-04 once and then incubated for 5 or 7 days or treated daily for 5 days at a concentration of 3:1 (Figures 9C–E).

#### 3.2.3. VH-04 possibly normalizes hippocampal *Syp* mRNA expression in aged rats

**Figure 10:**
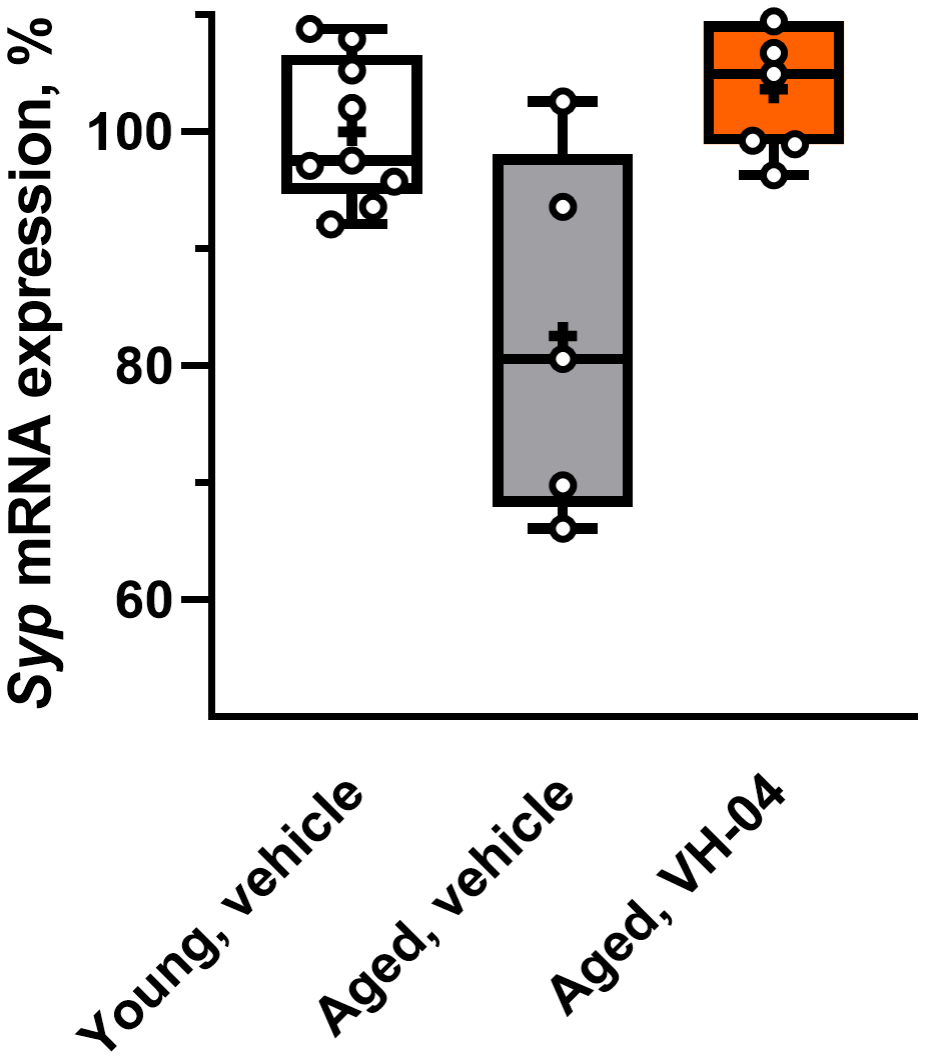
Effect of treatment with VH-04 on synaptophysin mRNA expression in hippocampal samples of aged rats. Expression levels were normalized by *Gapdh* mRNA levels. Data are presented as box-whisker plots (middle line: median; box: 25th and 75th percentiles; cross: mean value; whiskers: smallest and largest values; *N* = 5–9 in each group). Group factor significantly affected the relative *Syp* mRNA expression level [*F*_(2, 5.964)_ = 6.37; *p* = 0.0331, Brown-Forsythe ANOVA; see Supplementary Table 9 for the full breakdown of statistical results].

We harvested samples of hippocampus from a subset of aged rats used in Morris water maze experiments and ^1^H-MRS imaging to determine mRNA levels of the synaptic marker synaptophysin (Figure 10). Given that the Bartlett’s test indicated that standard deviations of *Syp* mRNA levels in the three groups were significantly different (corrected Bartlett’s statistic = 6.862, *p* = 0.0323), ordinary ANOVA could not be applied. Therefore, we utilized the Brown-Forsythe ANOVA, which is suitable for the analysis of groups with different variance and found that group factor significantly affected the level of *Syp* mRNA expression [*F*_(2, 5.964)_ = 6.37; *p* = 0.0331]. Although the Dunnett’s T3 multiple comparisons test did not reveal statistical differences between the groups in pairwise comparisons, we noted that the difference in the *Syp* mRNA level between vehicle- and VH-04-treated aged rats was nearly significant (*p* = 0.0847). This circumstance and significantly different standard deviations of *Syp* mRNA expression levels in the three groups suggest that vehicle-treated aged rats had a dissimilar pattern of *Syp* mRNA expression levels compared to that in vehicle-treated younger animals, whereas in VH-04-treated aged rats, this pattern was less apparent.

Thus, on the cellular and molecular levels, VH-04 promoted neurite outgrowth in mouse primary hippocampal neurons and possibly normalized hippocampal *Syp* mRNA expression in aged rats (Table 2).

**Table 2:** Summary of VH-04’s effects on biochemical and morphological parameters.

Parameter	Method	Species	Result
Age-related changes in hippocampal metabolic profile	^1^H-magnetic resonance spectroscopy	Rats	No effect
Aged-related synaptic changes inferred from hippocampal synaptophysin mRNA expression	RT-qPCR	Rats	Possible improvement: *p* = 0.0847 (vehicle- vs. VH-04-treated aged rats)
Primary hippocampal neurite elongation	NeuronJ plugin to measure neurite length	Mice	Improvement

## 4. Discussion

CI is triggered by various factors, including age, genetic variation, clinical status, environmental and social parameters. The efficacy of the available treatments to restore cognition or delay CI is limited (Fink et al., 2018), therefore the search for novel effective and safe therapeutics is continuing. Prompted by the available evidence of symptomatic improvements afforded by the administration of the multicomponent, multitarget drug VH-04 in preclinical models and patients with vertigo, we have examined the ability of VH-04 to improve performance of mice and rats in several cognitive tests. Our experiments demonstrated that treatment with VH-04 improved multiple types of memory in mice and rats. Our experiments also pointed to possible synaptic and cellular changes that could mediate these effects.

Most of the current clinically recommended drugs for CI have been developed on the premise of their known pharmacological effect on a single chosen enzyme or a receptor, e.g., cholinesterase antagonists, glutamate NMDA receptor blockers, or antibodies against amyloid β. This target-based approach has not yet produced therapeutics able to reverse cognitive decline completely, and the available positive effects are often transient and/or associated with gastrointestinal and cardiovascular side effects (Mimica and Presecki, 2009; Howes, 2014; Haake et al., 2020). Therefore, alternative interventions, such as plant extracts and nutraceuticals, are being actively tested in preclinical and clinical trials (Perry et al., 1998; Cicero et al., 2018; Howes et al., 2020; Ahmad et al., 2022). Notably, such supplementary and alternative medicines often contain multiple chemical entities and, therefore, able to engage a broader set of targets. Such multicomponent treatments have a long history of safe and effective usage in Eastern medicine, for example traditional Chinese medicine, ayurvedic and Kampo preparations (Wang et al., 2016; Takayama et al., 2020; Kuchta et al., 2021; Gayathri et al., 2022). Even in the case of single plant species, the overall effects on cognition may also be due to the diversity of chemical constituents and their multiple biological actions, as suggested, for example, for *Ginkgo biloba* and *Crocus sativus* (saffron) preparations (Sanaie et al., 2022; Villegas et al., 2022).

VH-04 is a multicomponent medicine used to treat vertigo, imbalance disorders and associated symptoms, such as nausea (Claussen et al., 1984; Zenner et al., 1991). The mechanisms of VH-04 effects are not well elucidated, but may involve a stimulating effect on the central nervous system activity either via vasodilatation or direct modulation of neurons. Administration of VH-04 modulated frequency components of EEG in a manner similar to that of cognition-enhancing tacrine and metanicotine as well as caffeine (Dimpfel et al., 2019) and seemed to improve cognitive performance in the eight-arm radial maze task of rats sham-treated for modeling vestibular syndrome (Hatat et al., 2022). These observations suggested that VH-04 may have cognition-enhancing activity.

Our present experiments demonstrated that administration of VH-04 indeed improved memory maintenance or alleviated the decline in rodent cognitive performance caused by the muscarinic antagonist scopolamine in several preclinical assays. Notably, VH-04 had a positive effect in the NOR test of visual recognition memory and ameliorated memory impairments induced by scopolamine in the spontaneous alternation test of working memory and the STFP test of olfactory memory, as did the established cognitive enhancer, cholinesterase inhibitor donepezil. Although both VH-04 and donepezil had a memory-improving effect in the spontaneous alternation test in mice, they both failed to enhance performance of scopolamine-treated rats in the rewarded alternation test. The reasons for this differential pharmacological sensitivity of the two related behavioral assays are not clear but they may be linked to the differences in the species, satiety status and experimental setting.

Furthermore, our experiments showed that repeated administration of VH-04 improved retention of spatial orientation memory in aged rats tested in the MWM. During the acquisition phase, the latency and distance to find the platform in vehicle- and VH-04-treated aged rats were significantly greater than in vehicle-treated younger counterparts, which confirmed previous reports about worse performance of older animals in the MWM (Gage et al., 1988; Deupree et al., 1991). Interestingly, although there were no differences between vehicle- and VH-04-treated aged rats in the course of the acquisition, during the probe trial, aged rats that received VH-04 remembered the location of the platform counter area whereas vehicle-treated aged rats did not.

A positive effect of VH-04 administration was also noted in the STFP test of olfactory memory, as mice that received 2 mL/kg VH-04 kept preferring cued food despite the treatment with scopolamine.

Cognitive performance in the NOR, spontaneous alternation, MWM and STFP tests depends on the structural integrity and activity of several brain areas, including the hippocampus, dorsal striatum, prefrontal cortex, piriform cortex, and others (Gage et al., 1988; D’Hooge and De Deyn, 2001; Alvarez et al., 2002; Lalonde, 2002; Sánchez-Andrade et al., 2005; Cohen and Stackman, 2015). Therefore, the stimulating effect of VH-04 may be mediated by the modulation of neuronal activity in many areas. One possible mechanism of such modulation is the upregulation of excitatory synaptic transmission, as the incubation with VH-04 was shown to augment population spikes in hippocampal slices (Dimpfel et al., 2019). The VH-04 component *Anamirta cocculus* could play a role in this effect, as it contains picrotoxin, a potent antagonist of type A γ-aminobutyric acid receptors (Van der Kloot and Robbins, 1959; Inoue and Akaike, 1988; Olsen, 2018). Picrotoxin administration improved performance of various genetically altered mice, including models of Alzheimer’s disease and Down syndrome, in novel object recognition, spontaneous alternation, and MWM tests (Fernandez et al., 2007; Yoshiike et al., 2008; Yu et al., 2013), although performance of control, wild-type animals usually remained unchanged (but see Na et al., 2014). At high doses, picrotoxin elicits seizures *in vivo* and evokes synchronous neuronal discharges in preparations *in vitro* due to potent disinhibition. Decades of safe use of VH-04 in patients demonstrate a good safety and tolerability profile of this medicine; no seizure-like adverse events have ever been reported following the treatment with VH-04.

In addition to picrotoxin, other constituents of VH-04 with potent pharmacological action are coniine and related piperidine alkaloids from *Conium maculatum*. Coniine is a neurotoxin and a teratogen, which at high doses blocks respiration and causes muscle paralysis by acting as a depolarizing neuromuscular blocker of nicotinic acetylcholine receptors (Schep et al., 2009; Green et al., 2010). However, these pharmacological effects become manifested at concentrations of coniine in the body that are several orders of magnitude higher than those present after exposure to VH-04. To the best of our knowledge, there have been no studies of the effects of coniine and other *Conium maculatum* alkaloids on cognitive behavior. Nonetheless, mild nicotinic acetylcholine receptor antagonism was demonstrated to prevent scopolamine-induced impairment of working memory in the spontaneous alternation task (Newman and Gold, 2016), facilitate learning in the eight-arm radial maze test (Levin and Caldwell, 2006) and improve recognition memory in individuals with attention-deficit/hyperactivity disorder (Potter et al., 2009).

The *in vitro* study performed by Heinle et al. (2010) using reconstituted (10,000×) VH-04 and single constituents (0–10^7^×) demonstrated concentration-dependent effects of the active VH-04 ingredients on adenylate cyclase and PDE5. Thus, the positive effects of VH-04 on cognitive behavior may be also associated with its mild stimulation of the cyclic nucleotide signaling pathways owing to the activation of adenylate cyclase activity and inhibition of PDE5 (Heinle et al., 2010). Treatment with forskolin that stimulates adenylate cyclase facilitated learning and memory in the MWM test (Kumar and Singh, 2017). In addition, agents that inhibited PDE5 improved performance in the NOR, MWM, STFP, and spontaneous alternation tests (Akar et al., 2014; Devan et al., 2014), which included restoration of scopolamine-induced memory impairments (Zhang et al., 2018; Tabrizian et al., 2021) and enhanced learning in Alzheimer’s disease models (Cuadrado-Tejedor et al., 2011; García-Barroso et al., 2013; Kang et al., 2022).

We have not observed a significant effect of VH-04 on the scopolamine-induced impairment of fear-aggravated memory in the passive avoidance test and memory for context in the fear conditioning test. As in the tests of spatial working memory, olfactory memory and visual recognition memory, the structural and functional integrity of the hippocampus plays a significant role in the execution of these tests. In addition, another important role in passive avoidance and fear conditioning is played by the amygdala (Kim and Jung, 2006). The fact that VH-04 decreased the scopolamine-induced memory impairment in the NOR, spatial alternation, and STFP tasks, which are partly hippocampus-dependent, but not in fear-aggravated paradigms, may indicate that scopolamine action is very strong in amygdala (Nomura et al., 1994; Wilson and Cook, 1994) and/or VH-04 does not engage targets in this brain area.

Our experiments showed that administration of VH-04 generally did not restore changes in brain metabolite levels in aged rats, as determined by ^1^H-MRS. The concentration of N-acetylaspartate, a metabolite that decreases with brain aging (Kirov et al., 2021), was numerically lower both in VH-04- and vehicle-treated aged rats than in young rats, but the difference was not statistically significant in the case of VH-04-treated animals. This could indicate a possible restorative action of the drug, but the extent of the effect was very small. Therefore, as the positive effect of VH-04 on spatial orientation memory in aged rats in the MWM was observed without pronounced changes in hippocampal metabolic profile, we hypothesize that this improvement could be attributed to changes in electrical activity in the hippocampus, as described earlier (Dimpfel et al., 2019).

It is generally believed that cognitive deficits in old age and in various brain disorders are often associated with the loss of synaptic integrity and structural plasticity in brain neurons (Urbanska et al., 2012; Radulescu et al., 2021; Walker and Herskowitz, 2021; Pham and Dore, 2022). Our experiments showed that 25-months-old rats treated with VH-04 had nominally higher mRNA expression of synaptophysin than vehicle-treated rats of the same age, although the effect did not reach statistical significance. Such synaptoprotective properties of VH-04 may be linked to its effect on cyclic nucleotide signaling, as the latter was demonstrated to increase synaptophysin expression and promote synapse formation/maintenance (Singh et al., 2020). Stimulating effects on mRNA and protein expression of synaptophysin and other synaptic markers were noted for other multicomponent preparations that were shown to improve learning and memory, such as Kampo formula Zokumei-to (Tohda et al., 2003), multi-herbal traditional Korean medicines Gami-Chunghyuldan (Choi et al., 2011) and PMC-12 (Park et al., 2016), and Goji berry extract (Ruíz-Salinas et al., 2020).

The capacity to remodel nerve endings may be important for learning and memory, and approaches to promote neurite length have been considered as a viable strategy of neuroprotection in neurodegenerative diseases and old age (Calabrese, 2008). In our experiments, a single exposure of mouse primary hippocampal neurons to VH-04 caused significant elongation of neurites in 5 and 7 days, indicating a neuroprotective potential of VH-04. A similar result was also observed after daily incubation of neurons with VH-04 for 5 days. This neurotrophic effect of VH-04 may be linked to its stimulating action on cyclic nucleotide signaling, as the latter is known to upregulate neurite outgrowth or prevent neurite retraction in primary neuronal cultures and cell lines (Wang and Zheng, 1998; Khodair et al., 2005; Stiles et al., 2014; Dar et al., 2020). In this regard, it is also interesting to note that neurite outgrowth is potentiated by other multicomponent preparations (Konaka et al., 2017; Terada et al., 2018; Chen et al., 2019; Xu et al., 2019; Chiu et al., 2021).

This study had several limitations. First, in many behavioral tests, the effect of VH-04 was estimated from its ability to decrease cognitive impairment following scopolamine-induced amnesia. Although it is a well-validated approach that has predictive validity in terms of cognition-improving effects, it is largely focused on the cholinergic system. Further preclinical experiments in genetic or other translational models of CI may provide additional information on the spectrum of VH-04 activity. Second, in some behavioral tests, e.g., fear conditioning (memory for the cue) and STFP, scopolamine produced minimal, if any, impairment, complicating the interpretation of VH-04 effects. Third, we explored the effect of VH-04 only on mRNA expression of one synaptic marker, whereas it would be valuable to obtain more detailed information about changes in both mRNA and protein expression levels of markers of excitatory and inhibitory synapses for a better elucidation of the mechanism of VH-04 action. Fourth, measurements of the drug’s effects on the learning performance alone and/or modulation of the targeted learning disruption (e.g., by scopolamine) do not reveal the actual molecular and cellular mechanisms of the drug’s action. These points will be addressed in future studies.

## 5. Conclusion

We examined the effects of VH-04 in behavioral tests in mice and rats and demonstrated that this multicomponent drug improved cognitive performance in the tests of visual recognition memory (NOR), spatial working memory (spontaneous alternation), spatial orientation memory (MWM) and olfactory memory (STFP). Experiments *in vitro* showed that VH-04 stimulated neurite growth and may protect synaptic integrity in aging brain. These properties allow a cautious conclusion that in addition to its ability to alleviate manifestations of vertigo, VH-04 may be used as a cognitive enhancer.

## Data availability statement

The raw data supporting the conclusions of this article will be made available by the authors, without undue reservation.

## Ethics statement

The animal study was reviewed and approved by the French Animal Health Regulations (Permit No. D 67–218-23 issued by the “Direction Départementale des Services Vétérinaires” of the Ministry of Agriculture and Fisheries) or experiments were carried out according to the National Institute of Health (NIH) guidelines for the care and use of laboratory animals and approved by the State Provincial Office of Southern Finland (license numbers 605/01/2006 and ESLH-2008-04752/Ym-23).

## Author contributions

BS, KO, YB, CS, AW, BE, and CA conceived the study. TH, KL, KP, JP, RP, EA, BH, SW, CS, AW, BE, and CA performed the experiments, analyzed, and interpreted the data. KW and BS supervised and coordinated the study. KW and BS wrote the original manuscript with the contributions from all authors. All authors contributed to the article and approved the submitted version.

## Funding

This study received funding from Heel GmbH, an international pharmaceutical company that specializes in developing and manufacturing medicines made from natural ingredients. The funder had the following involvement with the study: study design, provision of the tested substance, decision to publish, and preparation of the manuscript.

## Conflict of interest

Charles River Discovery Services Finland Oy and Neurofit are contract research organizations. At the time of the study, KW, KO, YB, and BS were employed by Heel GmbH. TH, KL, KP, JP, and RP were employed by Charles River Discovery Services Finland Oy. EA, BH, and SW were employed by Neurofit. CS, AW, BE, and CA were employed by the Ulm University. A patent application in relation to results has been filed as International Application no. PCT/EP2012/071898 (published as WO2013/068330) resulting in national patents and patent applications EP2776131, UA112784, RU2699038, and RU2019126669.

## Publisher’s note

All claims expressed in this article are solely those of the authors and do not necessarily represent those of their affiliated organizations, or those of the publisher, the editors and the reviewers. Any product that may be evaluated in this article, or claim that may be made by its manufacturer, is not guaranteed or endorsed by the publisher.
