# Supplementary material for: Vertigoheel promotes rodent cognitive performance in multiple memory tests
Source: Front Neurosci. 2023 May 31;17:1183023. doi: 10.3389/fnins.2023.1183023 (PMC10264630; doi:10.3389/fnins.2023.1183023)
Supplement: Supplementary file 1 [file Data_Sheet_1.docx]

Supplementary Material

# Supplementary Table 1. Statistical Comparisons for Figure 2

| Panel | Description | Test | Comparisons | Result | *P*-value |
| --- | --- | --- | --- | --- | --- |
| 1A | Discrimination index (time) | Ordinary one-way ANOVA  Post hoc Dunnett’s test | Group effect  "Saline, 24 h vs. Saline, 30 min"  "Saline, 24 h vs. VH-04, 0.1 mL/kg"  "Saline, 24 h vs. VH-04, 1 mL/kg"  "Saline, 24 h vs. VH-04, 2 mL/kg"  "Saline, 24 h vs. Donepezil, 0.3 mg/kg" | F_(5, 63)_ = 3.074  q = 3.555  q = 0.6740  q = 1.308  q = 2.317  q = 1.564 | 0.0152  0.0034  0.9428  0.5630  0.0932  0.3928 |
| 1B | Discrimination index (contact) | Ordinary one-way ANOVA  Post hoc Dunnett’s test | Group effect  "Saline, 24 h vs. Saline, 30 min"  "Saline, 24 h vs. VH-04, 0.1 mL/kg"  "Saline, 24 h vs. VH-04, 1 mL/kg"  "Saline, 24 h vs. VH-04, 2 mL/kg"  "Saline, 24 h vs. Donepezil, 0.3 mg/kg" | F_(5, 63)_ = 7.291  q = 5.209  q = 0.4171  q = 2.941  q = 3.084  q = 2.966 | <0.0001  <0.0001  0.9928  0.0199  0.0134  0.0185 |
| 1C | Distance run | Ordinary one-way ANOVA  Post hoc Dunnett’s test | Group effect  "Saline, 24 h vs. Saline, 30 min"  "Saline, 24 h vs. VH-04, 0.1 mL/kg"  "Saline, 24 h vs. VH-04, 1 mL/kg"  "Saline, 24 h vs. VH-04, 2 mL/kg"  "Saline, 24 h vs. Donepezil, 0.3 mg/kg" | F_(5, 63)_ = 4.511  q = 2.238  q = 1.195  q = 0.7916  q = 1.868  q = 1.900 | 0.0014  0.1109  0.6435  0.8962  0.2338  0.2205 |

# Supplementary Table 2. Statistical Comparisons for Figure 3

| Panel | Description | Test | Comparisons | Result | *P*-value |
| --- | --- | --- | --- | --- | --- |
| 2A | Spontaneous alternation, % | Ordinary one-way ANOVA  Post hoc Dunnett’s test | Group effect  "SCO vs. Vehicle"  "SCO vs. SCO + VH-04 (1 mL/kg single)"  "SCO vs. SCO + VH-04 (2 mL/kg single)"  "SCO (1 mg/kg) vs. SCO + VH-04 (1 mL/kg, q.d., 3 days)"  "SCO (1 mg/kg) vs. SCO + VH-04 (1 mL/kg, b.i.d., 3 days)"  "SCO (1 mg/kg) vs. SCO + donepezil" | F_(6, 63)_ = 16.61  q = 9.260  q = 6.704 q = 6.891 q = 7.087  q = 6.698  q = 6.701 | <0.0001  <0.0001  <0.0001 <0.0001 <0.0001  <0.0001  <0.0001 |
| 2B | Rewarded alternation, % | Ordinary one-way ANOVA  Post hoc Dunnett’s test | Group effect  "SCO vs. Vehicle"  "SCO vs. SCO + VH-04 (1 mL/kg single)"  "SCO vs. SCO + VH-04 (2 mL/kg single)"  "SCO (1 mg/kg) vs. SCO + VH-04 (1 mL/kg, q.d., 3 days)"  "SCO (1 mg/kg) vs. SCO + donepezil" | F_(5, 58)_ = 4.917  q = 3.776  q = 0.318  q = 0.383  q = 0.5223  q = 2.271 | 0.0008  0.0017  0.9971  0.9940  0.9762  0.0994 |

**Supplementary Table 3. Statistical Comparisons for Figure 4**

| Panel | Description | Test | Comparisons | Result | *P*-value |
| --- | --- | --- | --- | --- | --- |
| 3A | Latency, s | Two-way repeated measures ANOVA  Holm-Šídák's multiple comparisons test | Group effect  Trial effect  "Group × trial" interaction effect  *Trial 1*  "Aged, VH-04 vs. Aged, vehicle"  "Aged, VH-04 vs. Young, vehicle"  "Aged, vehicle vs. Young, vehicle"  *Trial 2*  "Aged, VH-04 vs. Aged, vehicle"  "Aged, VH-04 vs. Young, vehicle"  "Aged, vehicle vs. Young, vehicle"  *Trial 3*  "Aged, VH-04 vs. Aged, vehicle"  "Aged, VH-04 vs. Young, vehicle"  "Aged, vehicle vs. Young, vehicle"  *Trial 4*  "Aged, VH-04 vs. Aged, vehicle"  "Aged, VH-04 vs. Young, vehicle"  "Aged, vehicle vs. Young, vehicle"  *Trial 5*  "Aged, VH-04 vs. Aged, vehicle"  "Aged, VH-04 vs. Young, vehicle"  "Aged, vehicle vs. Young, vehicle"  *Trial 6*  "Aged, VH-04 vs. Aged, vehicle"  "Aged, VH-04 vs. Young, vehicle"  "Aged, vehicle vs. Young, vehicle"  *Trial 7*  "Aged, VH-04 vs. Aged, vehicle"  "Aged, VH-04 vs. Young, vehicle"  "Aged, vehicle vs. Young, vehicle"  *Trial 8*  "Aged, VH-04 vs. Aged, vehicle"  "Aged, VH-04 vs. Young, vehicle"  "Aged, vehicle vs. Young, vehicle"  *Trial 9*  "Aged, VH-04 vs. Aged, vehicle"  "Aged, VH-04 vs. Young, vehicle"  "Aged, vehicle vs. Young, vehicle"  *Trial 10*  "Aged, VH-04 vs. Aged, vehicle"  "Aged, VH-04 vs. Young, vehicle"  "Aged, vehicle vs. Young, vehicle  *Trial 11*  "Aged, VH-04 vs. Aged, vehicle"  "Aged, VH-04 vs. Young, vehicle"  "Aged, vehicle vs. Young, vehicle"  *Trial 12*  "Aged, VH-04 vs. Aged, vehicle"  "Aged, VH-04 vs. Young, vehicle"  "Aged, vehicle vs. Young, vehicle"  *Trial 13*  "Aged, VH-04 vs. Aged, vehicle"  "Aged, VH-04 vs. Young, vehicle"  "Aged, vehicle vs. Young, vehicle"  *Trial 14*  "Aged, VH-04 vs. Aged, vehicle"  "Aged, VH-04 vs. Young, vehicle"  "Aged, vehicle vs. Young, vehicle"  *Trial 15*  "Aged, VH-04 vs. Aged, vehicle"  "Aged, VH-04 vs. Young, vehicle"  "Aged, vehicle vs. Young, vehicle"  *Trial 16*  "Aged, VH-04 vs. Aged, vehicle"  "Aged, VH-04 vs. Young, vehicle"  "Aged, vehicle vs. Young, vehicle"  *Trial 17*  "Aged, VH-04 vs. Aged, vehicle"  "Aged, VH-04 vs. Young, vehicle"  "Aged, vehicle vs. Young, vehicle"  *Trial 18*  "Aged, VH-04 vs. Aged, vehicle"  "Aged, VH-04 vs. Young, vehicle"  "Aged, vehicle vs. Young, vehicle"  *Trial 19*  "Aged, VH-04 vs. Aged, vehicle"  "Aged, VH-04 vs. Young, vehicle"  "Aged, vehicle vs. Young, vehicle"  *Trial 20*  "Aged, VH-04 vs. Aged, vehicle"  "Aged, VH-04 vs. Young, vehicle"  "Aged, vehicle vs. Young, vehicle  *Trial 21*  "Aged, VH-04 vs. Aged, vehicle"  "Aged, VH-04 vs. Young, vehicle"  "Aged, vehicle vs. Young, vehicle"  *Trial 22*  "Aged, VH-04 vs. Aged, vehicle"  "Aged, VH-04 vs. Young, vehicle"  "Aged, vehicle vs. Young, vehicle"  *Trial 23*  "Aged, VH-04 vs. Aged, vehicle"  "Aged, VH-04 vs. Young, vehicle"  "Aged, vehicle vs. Young, vehicle"  *Trial 24*  "Aged, VH-04 vs. Aged, vehicle"  "Aged, VH-04 vs. Young, vehicle"  "Aged, vehicle vs. Young, vehicle"  *Trial 25*  "Aged, VH-04 vs. Aged, vehicle"  "Aged, VH-04 vs. Young, vehicle"  "Aged, vehicle vs. Young, vehicle"  *Trial 26*  "Aged, VH-04 vs. Aged, vehicle"  "Aged, VH-04 vs. Young, vehicle"  "Aged, vehicle vs. Young, vehicle"  *Trial 27*  "Aged, VH-04 vs. Aged, vehicle"  "Aged, VH-04 vs. Young, vehicle"  "Aged, vehicle vs. Young, vehicle"  *Trial 28*  "Aged, VH-04 vs. Aged, vehicle"  "Aged, VH-04 vs. Young, vehicle"  "Aged, vehicle vs. Young, vehicle"  *Trial 29*  "Aged, VH-04 vs. Aged, vehicle"  "Aged, VH-04 vs. Young, vehicle"  "Aged, vehicle vs. Young, vehicle"  *Trial 30*  "Aged, VH-04 vs. Aged, vehicle"  "Aged, VH-04 vs. Young, vehicle"  "Aged, vehicle vs. Young, vehicle  *Trial 31*  "Aged, VH-04 vs. Aged, vehicle"  "Aged, VH-04 vs. Young, vehicle"  "Aged, vehicle vs. Young, vehicle"  *Trial 33*  "Aged, VH-04 vs. Aged, vehicle"  "Aged, VH-04 vs. Young, vehicle"  "Aged, vehicle vs. Young, vehicle"  *Trial 33*  "Aged, VH-04 vs. Aged, vehicle"  "Aged, VH-04 vs. Young, vehicle"  "Aged, vehicle vs. Young, vehicle"  *Trial 34*  "Aged, VH-04 vs. Aged, vehicle"  "Aged, VH-04 vs. Young, vehicle"  "Aged, vehicle vs. Young, vehicle"  *Trial 35*  "Aged, VH-04 vs. Aged, vehicle"  "Aged, VH-04 vs. Young, vehicle"  "Aged, vehicle vs. Young, vehicle"  *Trial 36*  "Aged, VH-04 vs. Aged, vehicle"  "Aged, VH-04 vs. Young, vehicle"  "Aged, vehicle vs. Young, vehicle" | F_(2, 23)_ = 13.93  F_(11.54, 265.4)_ = 6.529  F_(70, 805)_ = 1.510  *t* = 0.6400  *t* = 0.7154  *t* = 1.439  *t* = 0.1502  *t* = 2.892  *t* = 3.257  *t* = 0.006675  *t* = 2.906  *t* = 2.807  *t* = 1.058  *t* = 5.729  *t* = 4.0  *t* = 0.01096  *t* = 1.877  *t* = 1.689  *t* = 0.5957  *t* = 2.538  *t* = 3.574  *t* = 0.2047  *t* = 3.961  *t* = 3.883  *t* = 0.2757  *t* = 2.535  *t* = 3.222  *t* = 0.7954  *t* = 5.682  *t* = 8.435  NA, as Δ = 0  *t* = 5.676  *t* = 5.676  *t* = 0.1378  *t* = 2.778  *t* = 3.318  *t* = 0.5896  *t* = 1.913  *t* = 2.721  *t* = 0.3873  *t* = 2.586  *t* = 1.836  *t* = 0.2201  *t* = 2.286  *t* = 2.057  *t* = 0.2653  *t* = 8.660  *t* = 5.817  *t* = 0.8202  *t* = 2.151  *t* = 1.358  *t* = 0.8223  *t* = 3.368  *t* = 2.485  *t* = 1.037  *t* = 3.324  *t* = 2.003  *t* = 0.5472  *t* = 2.467  *t* = 1.508  *t* = 1.649  *t* = 3.082  *t* = 1.181  *t* = 0.7746  *t* = 5.223  *t* = 2.951  *t* = 0.3949  *t* = 2.174  *t* = 2.693  *t* = 1.627  *t* = 3.764  *t* = 1.233  *t* = 0.03476  *t* = 0.05595  *t* = 0.01360  *t* = 0.2869  *t* = 3.429  *t* = 2.022  *t* = 0.1027  *t* = 0.4385  *t* = 0.4854  *t* = 0.5881  *t* = 2.257  *t* = 1.017  *t* = 0.4254  *t* = 2.936  *t* = 1.313  *t* = 1.110  *t* = 4.933  *t* = 1.753  *t* = 0.4818  *t* = 0.5457  *t* = 1.332  *t* = 0.7032  *t* = 1.895  *t* = 0.9946  0.09159  1.258  1.219  *t* = 0.8552  *t* = 3.115  *t* = 1.395  *t* = 0.2902  *t* = 0.7233  *t* = 0.2853  *t* = 0.6759  *t* = 2.034  *t* = 0.9702  *t* = 0.007188  *t* = 1.836  *t* = 1.582 | 0.0001  <0.0001  0.0058    0.7346  0.7346  0.4271  0.8829  0.0215  0.0171  0.9948  0.0308  0.0308  0.3100  0.0001  0.0021  0.9914  0.2189  0.2189  0.5616  0.0451  0.0076  0.8408  0.0095  0.0095  0.7869  0.0739  0.0403  0.4508  <0.0001  <0.0001  NA  NA  NA  0.8924  0.0506  0.0337  0.5648  0.1642  0.0642  0.7056  0.0632  0.1631  0.8289  0.1107  0.1139  0.7951  0.0001  0.0012  0.4262  0.1549  0.3580  0.4248  0.0316  0.0765  0.3174  0.0291  0.1501  0.5930  0.1183  0.3166  0.2294  0.0479  0.2725  0.4528  0.0014  0.0351  0.6988  0.1220  0.0833  0.2368  0.0152  0.2524  >0.9999  >0.9999  >0.9999  0.7790  0.0243  0.1523  0.9511  0.9511  0.9511  0.5665  0.1542  0.5654  0.6783  0.0609  0.4072  0.2896  0.0039  0.2284  0.8379  0.8379  0.5040  0.5791  0.2655  0.5791  0.9283  0.5685  0.5685  0.4077  0.0446  0.3652  0.9498  0.8625  0.9498  0.5930  0.2162  0.5930  0.9944  0.2839  0.2860 |
| 3B | Distance, cm | Two-way repeated measures ANOVA  Holm-Šídák's multiple comparisons test | Group effect  Trial effect  "Group × trial" interaction effect  *Trial 1*  "Aged, VH-04 vs. Aged, vehicle"  "Aged, VH-04 vs. Young, vehicle"  "Aged, vehicle vs. Young, vehicle"  *Trial 2*  "Aged, VH-04 vs. Aged, vehicle"  "Aged, VH-04 vs. Young, vehicle"  "Aged, vehicle vs. Young, vehicle"  *Trial 3*  "Aged, VH-04 vs. Aged, vehicle"  "Aged, VH-04 vs. Young, vehicle"  "Aged, vehicle vs. Young, vehicle"  *Trial 4*  "Aged, VH-04 vs. Aged, vehicle"  "Aged, VH-04 vs. Young, vehicle"  "Aged, vehicle vs. Young, vehicle"  *Trial 5*  "Aged, VH-04 vs. Aged, vehicle"  "Aged, VH-04 vs. Young, vehicle"  "Aged, vehicle vs. Young, vehicle"  *Trial 6*  "Aged, VH-04 vs. Aged, vehicle"  "Aged, VH-04 vs. Young, vehicle"  "Aged, vehicle vs. Young, vehicle"  *Trial 7*  "Aged, VH-04 vs. Aged, vehicle"  "Aged, VH-04 vs. Young, vehicle"  "Aged, vehicle vs. Young, vehicle"  *Trial 8*  "Aged, VH-04 vs. Aged, vehicle"  "Aged, VH-04 vs. Young, vehicle"  "Aged, vehicle vs. Young, vehicle"  *Trial 9*  "Aged, VH-04 vs. Aged, vehicle"  "Aged, VH-04 vs. Young, vehicle"  "Aged, vehicle vs. Young, vehicle"  *Trial 10*  "Aged, VH-04 vs. Aged, vehicle"  "Aged, VH-04 vs. Young, vehicle"  "Aged, vehicle vs. Young, vehicle  *Trial 11*  "Aged, VH-04 vs. Aged, vehicle"  "Aged, VH-04 vs. Young, vehicle"  "Aged, vehicle vs. Young, vehicle"  *Trial 12*  "Aged, VH-04 vs. Aged, vehicle"  "Aged, VH-04 vs. Young, vehicle"  "Aged, vehicle vs. Young, vehicle"  *Trial 13*  "Aged, VH-04 vs. Aged, vehicle"  "Aged, VH-04 vs. Young, vehicle"  "Aged, vehicle vs. Young, vehicle"  *Trial 14*  "Aged, VH-04 vs. Aged, vehicle"  "Aged, VH-04 vs. Young, vehicle"  "Aged, vehicle vs. Young, vehicle"  *Trial 15*  "Aged, VH-04 vs. Aged, vehicle"  "Aged, VH-04 vs. Young, vehicle"  "Aged, vehicle vs. Young, vehicle"  *Trial 16*  "Aged, VH-04 vs. Aged, vehicle"  "Aged, VH-04 vs. Young, vehicle"  "Aged, vehicle vs. Young, vehicle"  *Trial 17*  "Aged, VH-04 vs. Aged, vehicle"  "Aged, VH-04 vs. Young, vehicle"  "Aged, vehicle vs. Young, vehicle"  *Trial 18*  "Aged, VH-04 vs. Aged, vehicle"  "Aged, VH-04 vs. Young, vehicle"  "Aged, vehicle vs. Young, vehicle"  *Trial 19*  "Aged, VH-04 vs. Aged, vehicle"  "Aged, VH-04 vs. Young, vehicle"  "Aged, vehicle vs. Young, vehicle"  *Trial 20*  "Aged, VH-04 vs. Aged, vehicle"  "Aged, VH-04 vs. Young, vehicle"  "Aged, vehicle vs. Young, vehicle  *Trial 21*  "Aged, VH-04 vs. Aged, vehicle"  "Aged, VH-04 vs. Young, vehicle"  "Aged, vehicle vs. Young, vehicle"  *Trial 22*  "Aged, VH-04 vs. Aged, vehicle"  "Aged, VH-04 vs. Young, vehicle"  "Aged, vehicle vs. Young, vehicle"  *Trial 23*  "Aged, VH-04 vs. Aged, vehicle"  "Aged, VH-04 vs. Young, vehicle"  "Aged, vehicle vs. Young, vehicle"  *Trial 24*  "Aged, VH-04 vs. Aged, vehicle"  "Aged, VH-04 vs. Young, vehicle"  "Aged, vehicle vs. Young, vehicle"  *Trial 25*  "Aged, VH-04 vs. Aged, vehicle"  "Aged, VH-04 vs. Young, vehicle"  "Aged, vehicle vs. Young, vehicle"  *Trial 26*  "Aged, VH-04 vs. Aged, vehicle"  "Aged, VH-04 vs. Young, vehicle"  "Aged, vehicle vs. Young, vehicle"  *Trial 27*  "Aged, VH-04 vs. Aged, vehicle"  "Aged, VH-04 vs. Young, vehicle"  "Aged, vehicle vs. Young, vehicle"  *Trial 28*  "Aged, VH-04 vs. Aged, vehicle"  "Aged, VH-04 vs. Young, vehicle"  "Aged, vehicle vs. Young, vehicle"  *Trial 29*  "Aged, VH-04 vs. Aged, vehicle"  "Aged, VH-04 vs. Young, vehicle"  "Aged, vehicle vs. Young, vehicle"  *Trial 30*  "Aged, VH-04 vs. Aged, vehicle"  "Aged, VH-04 vs. Young, vehicle"  "Aged, vehicle vs. Young, vehicle  *Trial 31*  "Aged, VH-04 vs. Aged, vehicle"  "Aged, VH-04 vs. Young, vehicle"  "Aged, vehicle vs. Young, vehicle"  *Trial 33*  "Aged, VH-04 vs. Aged, vehicle"  "Aged, VH-04 vs. Young, vehicle"  "Aged, vehicle vs. Young, vehicle"  *Trial 33*  "Aged, VH-04 vs. Aged, vehicle"  "Aged, VH-04 vs. Young, vehicle"  "Aged, vehicle vs. Young, vehicle"  *Trial 34*  "Aged, VH-04 vs. Aged, vehicle"  "Aged, VH-04 vs. Young, vehicle"  "Aged, vehicle vs. Young, vehicle"  *Trial 35*  "Aged, VH-04 vs. Aged, vehicle"  "Aged, VH-04 vs. Young, vehicle"  "Aged, vehicle vs. Young, vehicle"  *Trial 36*  "Aged, VH-04 vs. Aged, vehicle"  "Aged, VH-04 vs. Young, vehicle"  "Aged, vehicle vs. Young, vehicle" | F_(2, 23)_ = 20.16  F_(11.70, 269)_ = 9.183  F_(70, 805)_ = 1.336  *t* = 0.6003  *t* = 0.7570  *t* = 0.3341  *t* =0.1369  *t* =0.1002  *t* =0.1893  *t* =0.3698  *t* =3.842  *t* =4.571  *t* =0.8177  *t* =0.6166  *t* =0.04998  *t* =0.04559  *t* =1.353  *t* =1.498  *t* =0.5609  *t* =1.643  *t* =2.262  *t* =0.7924  *t* =2.835  *t* =3.341  *t* =0.03030  *t* =1.463  *t* =1.359  *t* =0.1595  *t* =1.069  *t* =1.155  *t* =0.2739  *t* =3.069  *t* =3.138  *t* =0.3583  *t* =2.717  *t* =3.667  *t* =0.3985  *t* =7.476  *t* =6.317  *t* =0.5984  *t* =3.103  *t* =2.697  *t* =0.5467  *t* =2.366  *t* =2.896  *t* =0.5446  *t* =1.962  *t* =2.615  *t* =0.4723  *t* =1.868  *t* =1.307  *t* =0.2125  *t* =0.2473  *t* =0.4426  *t* =1.146  *t* =1.974  *t* =1.032  *t* =1.167  *t* =2.234  *t* =4.096  *t* =0.4720  *t* =2.989  *t* =3.716  *t* =0.09571  *t* =2.731  *t* =1.643  *t* =0.1452  *t* =2.640  *t* =2.412  *t* =0.9361  *t* =4.921  *t* =3.186  *t* =0.4829  *t* =2.624  *t* =3.831  *t* =1.435  *t* =2.305  *t* =0.6821  *t* =1.026  *t* =2.201  *t* =2.282  *t* =2.290  *t* =4.206  *t* =1.023  *t* =0.1002  *t* =3.899  *t* =2.029  *t* =2.024  *t* =5.504  *t* =3.366  *t* =0.1385  *t* =2.305  *t* =2.429  *t* =0.3893  *t* =1.605  *t* =1.700  *t* =0.4307  *t* =2.916  *t* =1.748  *t* =0.9217  *t* =3.662  *t* =5.430  *t* =0.2761  *t* =3.219  *t* =2.518  *t* =0.1401  *t* =3.139  *t* =3.082  *t* =1.445  *t* =1.539  *t* =2.526 | <0.0001  <0.0001    0.0393  0.8430  0.8430  0.8430  0.9968  0.9968  0.9968  0.7177  0.0030  0.0015  0.8137  0.8137  0.9608  0.9644  0.4068  0.4068  0.5840  0.2256  0.1113  0.4415  0.0289  0.0180  0.9763  0.4138  0.4138  0.8756  0.6044  0.6044  0.7883  0.0236  0.0236  0.7256  0.0526  0.0180  0.6963  0.0004  0.0007  0.5592  0.0206  0.0323  0.5932  0.0906  0.0616  0.5946  0.1468  0.0725  0.6440  0.2280  0.3799  0.9631  0.9631  0.9623  0.4699  0.1856  0.4699  0.2632  0.0959  0.0051  0.6442  0.0248  0.0098  0.9258  0.0441  0.2433  0.8867  0.0907  0.0907  0.3653  0.0032  0.0257  0.6369  0.0620  0.0150  0.3174  0.1020  0.5058  0.3306  0.1233  0.1233  0.0747  0.0074  0.3343  0.9221  0.0111  0.1521  0.0630  0.0018  0.0190  0.8919  0.1217  0.1217  0.7035  0.3149  0.3149  0.6736  0.0644  0.2315  0.3728  0.0082  0.0005  0.7865  0.0376  0.0731  0.8906  0.0482  0.0482  0.2808  0.2808  0.0998 |
| 3C | Speed, cm/s | Two-way repeated measures ANOVA  Holm-Šídák's multiple comparisons test | Group effect  Trial effect  "Group × trial" interaction effect  *Trial 1*  "Aged, VH-04 vs. Aged, vehicle"  "Aged, VH-04 vs. Young, vehicle"  "Aged, vehicle vs. Young, vehicle"  *Trial 2*  "Aged, VH-04 vs. Aged, vehicle"  "Aged, VH-04 vs. Young, vehicle"  "Aged, vehicle vs. Young, vehicle"  *Trial 3*  "Aged, VH-04 vs. Aged, vehicle"  "Aged, VH-04 vs. Young, vehicle"  "Aged, vehicle vs. Young, vehicle"  *Trial 4*  "Aged, VH-04 vs. Aged, vehicle"  "Aged, VH-04 vs. Young, vehicle"  "Aged, vehicle vs. Young, vehicle"  *Trial 5*  "Aged, VH-04 vs. Aged, vehicle"  "Aged, VH-04 vs. Young, vehicle"  "Aged, vehicle vs. Young, vehicle"  *Trial 6*  "Aged, VH-04 vs. Aged, vehicle"  "Aged, VH-04 vs. Young, vehicle"  "Aged, vehicle vs. Young, vehicle"  *Trial 7*  "Aged, VH-04 vs. Aged, vehicle"  "Aged, VH-04 vs. Young, vehicle"  "Aged, vehicle vs. Young, vehicle"  *Trial 8*  "Aged, VH-04 vs. Aged, vehicle"  "Aged, VH-04 vs. Young, vehicle"  "Aged, vehicle vs. Young, vehicle"  *Trial 9*  "Aged, VH-04 vs. Aged, vehicle"  "Aged, VH-04 vs. Young, vehicle"  "Aged, vehicle vs. Young, vehicle"  *Trial 10*  "Aged, VH-04 vs. Aged, vehicle"  "Aged, VH-04 vs. Young, vehicle"  "Aged, vehicle vs. Young, vehicle  *Trial 11*  "Aged, VH-04 vs. Aged, vehicle"  "Aged, VH-04 vs. Young, vehicle"  "Aged, vehicle vs. Young, vehicle"  *Trial 12*  "Aged, VH-04 vs. Aged, vehicle"  "Aged, VH-04 vs. Young, vehicle"  "Aged, vehicle vs. Young, vehicle"  *Trial 13*  "Aged, VH-04 vs. Aged, vehicle"  "Aged, VH-04 vs. Young, vehicle"  "Aged, vehicle vs. Young, vehicle"  *Trial 14*  "Aged, VH-04 vs. Aged, vehicle"  "Aged, VH-04 vs. Young, vehicle"  "Aged, vehicle vs. Young, vehicle"  *Trial 15*  "Aged, VH-04 vs. Aged, vehicle"  "Aged, VH-04 vs. Young, vehicle"  "Aged, vehicle vs. Young, vehicle"  *Trial 16*  "Aged, VH-04 vs. Aged, vehicle"  "Aged, VH-04 vs. Young, vehicle"  "Aged, vehicle vs. Young, vehicle"  *Trial 17*  "Aged, VH-04 vs. Aged, vehicle"  "Aged, VH-04 vs. Young, vehicle"  "Aged, vehicle vs. Young, vehicle"  *Trial 18*  "Aged, VH-04 vs. Aged, vehicle"  "Aged, VH-04 vs. Young, vehicle"  "Aged, vehicle vs. Young, vehicle"  *Trial 19*  "Aged, VH-04 vs. Aged, vehicle"  "Aged, VH-04 vs. Young, vehicle"  "Aged, vehicle vs. Young, vehicle"  *Trial 20*  "Aged, VH-04 vs. Aged, vehicle"  "Aged, VH-04 vs. Young, vehicle"  "Aged, vehicle vs. Young, vehicle  *Trial 21*  "Aged, VH-04 vs. Aged, vehicle"  "Aged, VH-04 vs. Young, vehicle"  "Aged, vehicle vs. Young, vehicle"  *Trial 22*  "Aged, VH-04 vs. Aged, vehicle"  "Aged, VH-04 vs. Young, vehicle"  "Aged, vehicle vs. Young, vehicle"  *Trial 23*  "Aged, VH-04 vs. Aged, vehicle"  "Aged, VH-04 vs. Young, vehicle"  "Aged, vehicle vs. Young, vehicle"  *Trial 24*  "Aged, VH-04 vs. Aged, vehicle"  "Aged, VH-04 vs. Young, vehicle"  "Aged, vehicle vs. Young, vehicle"  *Trial 25*  "Aged, VH-04 vs. Aged, vehicle"  "Aged, VH-04 vs. Young, vehicle"  "Aged, vehicle vs. Young, vehicle"  *Trial 26*  "Aged, VH-04 vs. Aged, vehicle"  "Aged, VH-04 vs. Young, vehicle"  "Aged, vehicle vs. Young, vehicle"  *Trial 27*  "Aged, VH-04 vs. Aged, vehicle"  "Aged, VH-04 vs. Young, vehicle"  "Aged, vehicle vs. Young, vehicle"  *Trial 28*  "Aged, VH-04 vs. Aged, vehicle"  "Aged, VH-04 vs. Young, vehicle"  "Aged, vehicle vs. Young, vehicle"  *Trial 29*  "Aged, VH-04 vs. Aged, vehicle"  "Aged, VH-04 vs. Young, vehicle"  "Aged, vehicle vs. Young, vehicle"  *Trial 30*  "Aged, VH-04 vs. Aged, vehicle"  "Aged, VH-04 vs. Young, vehicle"  "Aged, vehicle vs. Young, vehicle  *Trial 31*  "Aged, VH-04 vs. Aged, vehicle"  "Aged, VH-04 vs. Young, vehicle"  "Aged, vehicle vs. Young, vehicle"  *Trial 33*  "Aged, VH-04 vs. Aged, vehicle"  "Aged, VH-04 vs. Young, vehicle"  "Aged, vehicle vs. Young, vehicle"  *Trial 33*  "Aged, VH-04 vs. Aged, vehicle"  "Aged, VH-04 vs. Young, vehicle"  "Aged, vehicle vs. Young, vehicle"  *Trial 34*  "Aged, VH-04 vs. Aged, vehicle"  "Aged, VH-04 vs. Young, vehicle"  "Aged, vehicle vs. Young, vehicle"  *Trial 35*  "Aged, VH-04 vs. Aged, vehicle"  "Aged, VH-04 vs. Young, vehicle"  "Aged, vehicle vs. Young, vehicle"  *Trial 36*  "Aged, VH-04 vs. Aged, vehicle"  "Aged, VH-04 vs. Young, vehicle"  "Aged, vehicle vs. Young, vehicle" | F_(2, 23)_ = 1.167  F_(12, 275.9)_ = 3.964  F_(70, 805)_ = 2.662  *t* =0.1881  *t* =4.677  *t* =4.478  *t* =0.9785  *t* =5.450  *t* =2.813  *t* =0.3759  *t* =1.492  *t* =1.745  *t* =0.2872  *t* =3.650  *t* =3.057  *t* =0.1314  *t* =3.125  *t* =3.573  *t* =0.1513  *t* =1.182  *t* =1.233  *t* =0.7876  *t* =2.524  *t* =1.911  *t* =0.6243  *t* =1.127  *t* =1.948  *t* =0.3761  *t* =2.790  *t* =1.896  *t* =0.03743  *t* =1.245  *t* =1.177  *t* =0.4185  *t* =0.6319  *t* =1.010  *t* =0.2251  *t* =1.053  *t* =0.7123  *t* =0.4630  *t* =1.299  *t* =0.7483  *t* =1.434  *t* =0.1204  *t* =1.327  *t* =0.8515  *t* =0.5032  *t* =0.3085  *t* =0.5988  *t* =0.05913  *t* =0.7061  *t* =0.5371  *t* =1.751  *t* =2.355  *t* =0.2271  *t* =1.845  *t* =1.399  *t* =0.09278  *t* =1.008  *t* =1.323  *t* =0.07789  *t* =1.234  *t* =1.524  *t* =1.071  *t* =1.793  *t* =0.4414  *t* =0.2655  *t* =0.6395  *t* =0.8180  *t* =0.3958  *t* =2.483  *t* =1.839  *t* =0.3554  *t* =0.8780  *t* =0.4054  *t* =0.9349  *t* =1.818  *t* =0.3049  *t* =1.083  *t* =1.321  *t* =0.2415  *t* =0.09516  *t* =1.055  *t* =1.402  *t* =0.5371  *t* =0.04584  *t* =0.5853  *t* =0.9431  *t* =1.854  *t* =0.2508  *t* =0.2453  *t* =0.9680  *t* =0.6666  *t* =0.1289  *t* =0.5038  *t* =0.3832  *t* =0.9914  *t* =0.3772  *t* =0.8171  *t* =0.08355  *t* =0.1315  *t* =0.04543  *t* =0.2913  *t* =0.7100  *t* =0.5609  *t* =0.1810  *t* =0.3853  *t* =0.2508  *t* =0.3875  *t* =0.1969  *t* =0.7273 | 0.3291  <0.0001    <0.0001  0.8535  0.0008  0.0008  0.3501  0.0002  0.0305  0.7126  0.2901  0.2752  0.7792  0.0066  0.0171  0.8973  0.0199  0.0123  0.8820  0.5536  0.5536  0.4443  0.0662  0.1430  0.5444  0.4767  0.1991  0.7129  0.0425  0.1593  0.9707  0.5471  0.5471  0.7851  0.7851  0.6962  0.8253  0.6709  0.7386  0.7148  0.5121  0.7148  0.4384  0.9057  0.4384  0.7937  0.8569  0.8569  0.8676  0.9536  0.8676  0.5996  0.1899  0.0929  0.8238  0.2307  0.3317  0.9276  0.5501  0.4980  0.9391  0.4192  0.3803  0.5139  0.2580  0.6664  0.8109  0.8109  0.8109  0.6984  0.0731  0.1666  0.9050  0.7776  0.9050  0.6005  0.2535  0.7669  0.5069  0.5017  0.8123  0.9259  0.5305  0.4507  0.9204  0.9642  0.9204  0.6025  0.2289  0.8064  0.8098  0.7294  0.7680  0.9469  0.9469  0.9469  0.7117  0.7126  0.7117  0.9989  0.9989  0.9989  0.8717  0.8717  0.8717  0.9750  0.9750  0.9750  0.9127  0.9127  0.8584 |
| 3D | Thigmotaxis, % | Two-way repeated measures ANOVA  Holm-Šídák's multiple comparisons test | Group effect  Trial effect  "Group × trial" interaction effect  *Trial 1*  "Aged, VH-04 vs. Aged, vehicle"  "Aged, VH-04 vs. Young, vehicle"  "Aged, vehicle vs. Young, vehicle"  *Trial 2*  "Aged, VH-04 vs. Aged, vehicle"  "Aged, VH-04 vs. Young, vehicle"  "Aged, vehicle vs. Young, vehicle"  *Trial 3*  "Aged, VH-04 vs. Aged, vehicle"  "Aged, VH-04 vs. Young, vehicle"  "Aged, vehicle vs. Young, vehicle"  *Trial 4*  "Aged, VH-04 vs. Aged, vehicle"  "Aged, VH-04 vs. Young, vehicle"  "Aged, vehicle vs. Young, vehicle"  *Trial 5*  "Aged, VH-04 vs. Aged, vehicle"  "Aged, VH-04 vs. Young, vehicle"  "Aged, vehicle vs. Young, vehicle"  *Trial 6*  "Aged, VH-04 vs. Aged, vehicle"  "Aged, VH-04 vs. Young, vehicle"  "Aged, vehicle vs. Young, vehicle"  *Trial 7*  "Aged, VH-04 vs. Aged, vehicle"  "Aged, VH-04 vs. Young, vehicle"  "Aged, vehicle vs. Young, vehicle"  *Trial 8*  "Aged, VH-04 vs. Aged, vehicle"  "Aged, VH-04 vs. Young, vehicle"  "Aged, vehicle vs. Young, vehicle"  *Trial 9*  "Aged, VH-04 vs. Aged, vehicle"  "Aged, VH-04 vs. Young, vehicle"  "Aged, vehicle vs. Young, vehicle"  *Trial 10*  "Aged, VH-04 vs. Aged, vehicle"  "Aged, VH-04 vs. Young, vehicle"  "Aged, vehicle vs. Young, vehicle  *Trial 11*  "Aged, VH-04 vs. Aged, vehicle"  "Aged, VH-04 vs. Young, vehicle"  "Aged, vehicle vs. Young, vehicle"  *Trial 12*  "Aged, VH-04 vs. Aged, vehicle"  "Aged, VH-04 vs. Young, vehicle"  "Aged, vehicle vs. Young, vehicle"  *Trial 13*  "Aged, VH-04 vs. Aged, vehicle"  "Aged, VH-04 vs. Young, vehicle"  "Aged, vehicle vs. Young, vehicle"  *Trial 14*  "Aged, VH-04 vs. Aged, vehicle"  "Aged, VH-04 vs. Young, vehicle"  "Aged, vehicle vs. Young, vehicle"  *Trial 15*  "Aged, VH-04 vs. Aged, vehicle"  "Aged, VH-04 vs. Young, vehicle"  "Aged, vehicle vs. Young, vehicle"  *Trial 16*  "Aged, VH-04 vs. Aged, vehicle"  "Aged, VH-04 vs. Young, vehicle"  "Aged, vehicle vs. Young, vehicle"  *Trial 17*  "Aged, VH-04 vs. Aged, vehicle"  "Aged, VH-04 vs. Young, vehicle"  "Aged, vehicle vs. Young, vehicle"  *Trial 18*  "Aged, VH-04 vs. Aged, vehicle"  "Aged, VH-04 vs. Young, vehicle"  "Aged, vehicle vs. Young, vehicle"  *Trial 19*  "Aged, VH-04 vs. Aged, vehicle"  "Aged, VH-04 vs. Young, vehicle"  "Aged, vehicle vs. Young, vehicle"  *Trial 20*  "Aged, VH-04 vs. Aged, vehicle"  "Aged, VH-04 vs. Young, vehicle"  "Aged, vehicle vs. Young, vehicle  *Trial 21*  "Aged, VH-04 vs. Aged, vehicle"  "Aged, VH-04 vs. Young, vehicle"  "Aged, vehicle vs. Young, vehicle"  *Trial 22*  "Aged, VH-04 vs. Aged, vehicle"  "Aged, VH-04 vs. Young, vehicle"  "Aged, vehicle vs. Young, vehicle"  *Trial 23*  "Aged, VH-04 vs. Aged, vehicle"  "Aged, VH-04 vs. Young, vehicle"  "Aged, vehicle vs. Young, vehicle"  *Trial 24*  "Aged, VH-04 vs. Aged, vehicle"  "Aged, VH-04 vs. Young, vehicle"  "Aged, vehicle vs. Young, vehicle"  *Trial 25*  "Aged, VH-04 vs. Aged, vehicle"  "Aged, VH-04 vs. Young, vehicle"  "Aged, vehicle vs. Young, vehicle"  *Trial 26*  "Aged, VH-04 vs. Aged, vehicle"  "Aged, VH-04 vs. Young, vehicle"  "Aged, vehicle vs. Young, vehicle"  *Trial 27*  "Aged, VH-04 vs. Aged, vehicle"  "Aged, VH-04 vs. Young, vehicle"  "Aged, vehicle vs. Young, vehicle"  *Trial 28*  "Aged, VH-04 vs. Aged, vehicle"  "Aged, VH-04 vs. Young, vehicle"  "Aged, vehicle vs. Young, vehicle"  *Trial 29*  "Aged, VH-04 vs. Aged, vehicle"  "Aged, VH-04 vs. Young, vehicle"  "Aged, vehicle vs. Young, vehicle"  *Trial 30*  "Aged, VH-04 vs. Aged, vehicle"  "Aged, VH-04 vs. Young, vehicle"  "Aged, vehicle vs. Young, vehicle  *Trial 31*  "Aged, VH-04 vs. Aged, vehicle"  "Aged, VH-04 vs. Young, vehicle"  "Aged, vehicle vs. Young, vehicle"  *Trial 33*  "Aged, VH-04 vs. Aged, vehicle"  "Aged, VH-04 vs. Young, vehicle"  "Aged, vehicle vs. Young, vehicle"  *Trial 33*  "Aged, VH-04 vs. Aged, vehicle"  "Aged, VH-04 vs. Young, vehicle"  "Aged, vehicle vs. Young, vehicle"  *Trial 34*  "Aged, VH-04 vs. Aged, vehicle"  "Aged, VH-04 vs. Young, vehicle"  "Aged, vehicle vs. Young, vehicle"  *Trial 35*  "Aged, VH-04 vs. Aged, vehicle"  "Aged, VH-04 vs. Young, vehicle"  "Aged, vehicle vs. Young, vehicle"  *Trial 36*  "Aged, VH-04 vs. Aged, vehicle"  "Aged, VH-04 vs. Young, vehicle"  "Aged, vehicle vs. Young, vehicle" | F_(2, 23)_ = 8.54  F_(7.547, 173.6)_ = 4.681  F_(70, 805)_ = 2.086  *t* =0.2795  *t* =1.486  *t* =1.322  *t* =0.2217  *t* =4.426  *t* =3.753  *t* =0.9715  *t* =5.091  *t* =12.43  *t* =0.3164  *t* =4.842  *t* =4.412  *t* =1.099  *t* =3.977  *t* =3.615  *t* =0.1960  *t* =2.956  *t* =2.892  *t* =1.336  *t* =4.781  *t* =4.524  *t* =0.3302  *t* =2.113  *t* =2.572  *t* =0.01891  *t* =4.119  *t* =7.206  *t* =0.6186  *t* =3.070  *t* =3.019  *t* =0.2361  *t* =3.135  *t* =2.982  *t* =0.1795  *t* =4.470  *t* =3.243  *t* =0.7506  *t* =4.949  *t* =3.532  *t* =0.1473  *t* =2.002  *t* =2.547  *t* =0.4903  *t* =3.732  *t* =2.999  *t* =0.06563  *t* =3.086  *t* =2.297  *t* =0.3148  *t* =2.532  *t* =2.485  *t* =0.6296  *t* =2.708  *t* =2.003  *t* =0.6544  *t* =2.988  *t* =1.508  *t* =1.095  *t* =1.931  *t* =1.181  *t* =0.006828  *t* =2.058  *t* =2.951  *t* =0.4904  *t* =2.337  *t* =2.693  *t* =0.03434  *t* =1.434  *t* =1.233  *t* =1.285  *t* =1.465  *t* =0.01360  *t* =0.2519  *t* =2.325  *t* =2.022  *t* =0.8511  *t* =0.2860  *t* =0.4854  *t* =0.3713  *t* =0.7455  *t* =1.017  *t* =0.02803  *t* =2.228  *t* =1.313  *t* =0.5852  *t* =2.074  *t* =1.753  *t* =0.3257  *t* =2.338  *t* =1.332  *t* =1.212  *t* =2.565  *t* =0.9946  *t* =0.3717  *t* =1.455  *t* =1.219  *t* =0.1649  *t* =2.583  *t* =1.395  *t* =1.005  *t* =1.291  *t* =0.2853  *t* =0.6002  *t* =1.751  *t* =0.9702  *t* =0.2542  *t* =1.627  *t* =1.582 | 0.0017  <0.0001    <0.0001  0.7841  0.4099  0.4099  0.8280  0.0032  0.0087  0.3570  0.0014  <0.0001  0.7564  0.0007  0.0011  0.2930  0.0045  0.0047  0.8475  0.0310  0.0310  0.2062  0.0013  0.0013  0.7462  0.1080  0.0688  0.9853  0.0052  <0.0001  0.5472  0.0263  0.0263  0.8168  0.0393  0.0393  0.8603  0.0067  0.0259  0.4655  0.0015  0.0113  0.8851  0.1512  0.0917  0.6315  0.0176  0.0345  0.9487  0.0364  0.0962  0.7577  0.1071  0.1071  0.5392  0.0757  0.1501  0.5242  0.0544  0.3166  0.4708  0.2548  0.4708  0.9947  0.1452  0.0521  0.6316  0.0942  0.0833  0.9731  0.4650  0.4650  0.4473  0.4473  0.9894  0.8048  0.1425  0.1523  0.7970  0.8662  0.8662  0.7269  0.7269  0.7135  0.9781  0.1678  0.4072  0.5708  0.2010  0.2284  0.7506  0.0953  0.3734  0.4310  0.1033  0.4310  0.7160  0.4618  0.4618  0.8719  0.0927  0.3652  0.5693  0.5202  0.7811  0.5930  0.3196  0.5930  0.8032  0.3767  0.3767 |
| 3E | Time in quadrant, % | Two-way repeated measures ANOVA  Holm-Šídák's multiple comparisons test | Group effect  Quadrant effect "Group × quadrant" interaction effect  *Aged, VH-04*  " Quadrant 1 vs. Quadrant 2"  " Quadrant 1 vs. Quadrant 3"  " Quadrant 1 vs. Quadrant 4"  " Quadrant 2 vs. Quadrant 3"  " Quadrant 2 vs. Quadrant 4"  " Quadrant 3 vs. Quadrant 4"  *Aged, vehicle*  " Quadrant 1 vs. Quadrant 2"  " Quadrant 1 vs. Quadrant 3"  " Quadrant 1 vs. Quadrant 4"  " Quadrant 2 vs. Quadrant 3"  " Quadrant 2 vs. Quadrant 4"  " Quadrant 3 vs. Quadrant 4"  *Young, vehicle*  " Quadrant 1 vs. Quadrant 2"  " Quadrant 1 vs. Quadrant 3"  " Quadrant 1 vs. Quadrant 4"  " Quadrant 2 vs. Quadrant 3"  " Quadrant 2 vs. Quadrant 4"  " Quadrant 3 vs. Quadrant 4"" | F_(2, 23)_ = 0.9794  F_(3, 69)_ = 42.94  F_(6, 69)_ = 2.641  *t* =0.8826  *t* =4.809  *t* =1.170  *t* =3.927  *t* =0.2869  *t* =3.640  *t* =0.8291  *t* =4.792  *t* =1.916  *t* =3.963  *t* =1.087  *t* =2.876  *t* =1.642  *t* =7.762  *t* =1.886  *t* =9.404  *t* =3.527  *t* =5.876 | 0.3907  <0.0001  0.023  0.6162  <0.0001  0.5717  0.0010  0.7750  0.0021  0.4829  <0.0001  0.1681  0.0009  0.4829  0.0212  0.1231  <0.0001  0.1231  <0.0001  0.0023  <0.0001 |
| 3F | Passes through counter | Two-way repeated measures ANOVA  Holm-Šídák's multiple comparisons test | Group effect  Quadrant effect "Group × quadrant" interaction effect  *Aged, VH-04*  " Quadrant 1 vs. Quadrant 2"  " Quadrant 1 vs. Quadrant 3"  " Quadrant 1 vs. Quadrant 4"  " Quadrant 2 vs. Quadrant 3"  " Quadrant 2 vs. Quadrant 4"  " Quadrant 3 vs. Quadrant 4"  *Aged, vehicle*  " Quadrant 1 vs. Quadrant 2"  " Quadrant 1 vs. Quadrant 3"  " Quadrant 1 vs. Quadrant 4"  " Quadrant 2 vs. Quadrant 3"  " Quadrant 2 vs. Quadrant 4"  " Quadrant 3 vs. Quadrant 4"  *Young, vehicle*  " Quadrant 1 vs. Quadrant 2"  " Quadrant 1 vs. Quadrant 3"  " Quadrant 1 vs. Quadrant 4"  " Quadrant 2 vs. Quadrant 3"  " Quadrant 2 vs. Quadrant 4"  " Quadrant 3 vs. Quadrant 4"" | F_(2, 23)_ = 11.77  F_(3, 69)_ = 24.30  F_(6, 69)_ = 4.46  *t* =0.2620  *t* =3.668  *t* =0.7860  *t* =3.406  *t* =0.5240  *t* =2.882  *t* =0.2620  *t* =1.572  *t* =0.2620  *t* =1.310  *t* =0.000  *t* =1.310  *t* =0.4687  *t* =7.733  *t* =0.7030  *t* =8.202  *t* =1.172  *t* =7.030 | 0.0003  <0.0001  0.0007  0.8416  0.0029  0.8192  0.0055  0.8416  0.0209  0.9913  0.5373  0.9913  0.6610  >0.9999  0.6610  0.7342  <0.0001  0.7342  <0.0001  0.5702  <0.0001 |
| 3G | Time in counter, % | Two-way repeated measures ANOVA  Holm-Šídák's multiple comparisons test | Group effect  Quadrant effect "Group × quadrant" interaction effect  *Aged, VH-04*  " Quadrant 1 vs. Quadrant 2"  " Quadrant 1 vs. Quadrant 3"  " Quadrant 1 vs. Quadrant 4"  " Quadrant 2 vs. Quadrant 3"  " Quadrant 2 vs. Quadrant 4"  " Quadrant 3 vs. Quadrant 4"  *Aged, vehicle*  " Quadrant 1 vs. Quadrant 2"  " Quadrant 1 vs. Quadrant 3"  " Quadrant 1 vs. Quadrant 4"  " Quadrant 2 vs. Quadrant 3"  " Quadrant 2 vs. Quadrant 4"  " Quadrant 3 vs. Quadrant 4"  *Young, vehicle*  " Quadrant 1 vs. Quadrant 2"  " Quadrant 1 vs. Quadrant 3"  " Quadrant 1 vs. Quadrant 4"  " Quadrant 2 vs. Quadrant 3"  " Quadrant 2 vs. Quadrant 4"  " Quadrant 3 vs. Quadrant 4"" | F_(2, 23)_ = 5.664  F_(3, 69)_ = 31.5  F_(6, 69)_ = 4.385  *t* =0.1253  *t* =4.324  *t* =0.8773  *t* =4.198  *t* =0.7519  *t* =3.446  *t* =0.5431  *t* =2.151  *t* =0.1671  *t* =1.608  *t* =0.3760  *t* =1.984  *t* =0.4297  *t* =8.201  *t* =0.5044  *t* =8.631  *t* =0.9341  *t* =7.697 | 0.01  <0.0001  0.0008  0.9006  0.0003  0.7656  0.0004  0.7656  0.0039  0.9305  0.1922  0.9305  0.3791  0.9305  0.2311  0.8522  <0.0001  0.8522  <0.0001  0.7298  <0.0001 |

# Supplementary Table 4. Statistical Comparisons for Figure 5

| Panel | Description | Test | Comparisons | Result | *P*-value |
| --- | --- | --- | --- | --- | --- |
| Day 1 | Latency to enter dark chamber, s | Ordinary one-way ANOVA | Group effect | F_(5, 88)_ = 0.893 | 0.4895 |
| Day 2 | Latency to enter dark chamber, s | Ordinary one-way ANOVA  Post hoc Dunnett’s test | Group effect  "SCO vs. Vehicle"  "SCO vs. SCO + VH-04 (2 mL/kg single)"  "SCO vs. SCO + VH-04 (1 mL/kg, q.d., 3 days)"  "SCO vs. SCO + VH-04 (2 mL/kg, q.d., 3 days)"  "SCO vs. SCO + donepezil" | F_(5, 88)_ = 7.078  q = 5.239  q = 1.995  q = 0.9418  q = 0.4257  q = 1.5 | <0.0001  <0.0001  0.1793  0.8132  0.9920  0.4284 |

# Supplementary Table 5. Statistical Comparisons for Figure 6

| Panel | Description | Test | Comparisons | Result | *P*-value |
| --- | --- | --- | --- | --- | --- |
| 5A | Freezing to context, % | Ordinary one-way ANOVA  Post hoc Dunnett’s test | Group effect  "SCO vs. Vehicle"  "SCO vs. SCO + VH-04 (1 mL/kg single)"  "SCO vs. SCO + VH-04 (2 mL/kg, single)"  "SCO vs. SCO + VH-04 (1 mL/kg, q.d., 3 days)"  "SCO vs. SCO + donepezil" | F_(5, 81)_ = 3.403  q = 3.259  q = 0.4311  q = 0.2373  q = 1.309  q = 1.681 | 0.0077  0.0074  0.9916  0.9996  0.5603  0.3219 |
| 5B | Freezing to altered context, % | Ordinary one-way ANOVA | Group effect | F_(5, 88)_ = 0.9495 | 0.4539 |
| 5C | Freezing to cue, % | Ordinary one-way ANOVA | Group effect | F_(5, 88)_ = 1.426 | 0.2239 |

# Supplementary Table 6. Statistical Comparisons for Figure 7

| Description | Test | Comparisons | Result | *P*-value |
| --- | --- | --- | --- | --- |
| Amount of food, g | Ordinary two-way ANOVA  Post hoc Holm-Šídák's multiple comparisons test | Group effect  Food type effect  “Group × food type” interaction effect  Cued vs. non-cued within each group:  Vehicle  SCO  SCO + VH-04 (1 mL/kg single)  SCO + VH-04 (2 mL/kg, single)  SCO + VH-04 (1 mL/kg, q.d., 3 days)  SCO + donepezil | F_(5, 156)_ = 0.132  F_(1, 156)_ = 39.39  F_(1, 156)_ = 2.818  *t* = 4.177  *t* = 2.311  *t* = 0.1015  *t* = 2.558  *t* = 1.550  *t* = 4.910 | 0.9848  <0.0001  0.0182  0.0002  0.0650  0.9193  0.0452  0.2313  <0.0001 |

# Supplementary Table 7. Statistical Comparisons for Figure 8

| Description | Test | Comparisons | Result | *P*-value |
| --- | --- | --- | --- | --- |
| Concentration, mmol/kg | Ordinary two-way ANOVA  Post hoc Holm-Šídák's multiple comparisons test | **Creatine (CR)**  Group effect  "Young, vehicle vs. Aged, vehicle"  "Young, vehicle vs. Aged, VH-04"  "Aged, vehicle vs. Aged, VH-04"  **Phosphocreatine (PCR)**  Group effect  **Glutamine (GLN)**  Group effect  **Glutamate (GLU)**  Group effect  "Young, vehicle vs. Aged, vehicle"  "Young, vehicle vs. Aged, VH-04"  "Aged, vehicle vs. Aged, VH-04"  **Inositol (INS)**  Group effect  "Young, vehicle vs. Aged, vehicle"  "Young, vehicle vs. Aged, VH-04"  "Aged, vehicle vs. Aged, VH-04"  ***N*-acetyl-aspartate (NAA)**  Group effect  "Young, vehicle vs. Aged, vehicle"  "Young, vehicle vs. Aged, VH-04"  "Aged, vehicle vs. Aged, VH-04"  **Taurine (TAU)**  Group effect  **Choline (CHO)**  Group effect  **Choline (CHO)**  Group effect  ***N*-acetyl-aspartate + *N*-acetyl-aspartyl-glutamate (NAA + NAAG)**  Group effect  **Creatine + phosphocreatine (CR + PCR)**  Group effect  **Glutamate + glutamine (GLU + GLN)**  Group effect | F_(2, 23)_ = 6.046  *t* = 3.095  *t* = 2.795  *t* = 0.2849  F_(2, 23)_ = 2.615  F_(2, 23)_ = 0.6439  F_(2, 23)_ = 4.975  *t* = 2.669  *t* = 2.692  *t* = 0.02217  F_(2, 23)_ = 18.24  *t* = 4.210  *t* = 5.750  *t* = 1.461  F_(2, 23)_ = 4.227  *t* = 2.635  *t* = 2.272  *t* = 0.3445  F_(2, 23)_ = 2.257  F_(2, 23)_ = 0.2934  F_(2, 23)_ = 2.663  F_(2, 23)_ = 0.2757  F_(2, 23)_ = 3.371 | 0.0078  0.0152  0.0205  0.7783  0.0948  0.4487  0.016  0.0385  0.0385  0.9825  <0.0001  0.0007  <0.0001  0.1576  0.0273  0.0437  0.0644  0.7336  0.1274  0.7485  0.0911  0.7615  0.0520 |

# Supplementary Table 8. Statistical Comparisons for Figure 9

| Panel | Description | Test | Comparisons | Result | *P*-value |
| --- | --- | --- | --- | --- | --- |
| A | Elongation of neurites, μm | Unpaired *t*-test | Vehicle vs. VH-04, 1:3, 3 days | *t*_(38)_ = 0.6077 | 0.547 |
| B | Elongation of neurites, μm | Unpaired *t*-test | Vehicle vs. VH-04, 1:4, 3 days | *t*_(37)_ = 1.023 | 0.3132 |
| C | Elongation of neurites, μm | Unpaired *t*-test with Welch’s correction | Vehicle vs. VH-04, 3:1, 5 days | *t*_(68.96)_ = 2.01 | 0.0484 |
| D | Elongation of neurites, μm | Unpaired *t*-test with Welch’s correction | Vehicle vs. VH-04, 3:1, 5 days, daily treatment | *t*_(64.65)_ = 2.503 | 0.0149 |
| E | Elongation of neurites, μm | Unpaired *t*-test | Vehicle vs. VH-04, 3:1, 7 days | *t*_(138)_ = 4.048 | <0.0001 |

# Supplementary Table 9. Statistical Comparisons for Figure 10

| Description | Test | Comparisons | Result | *P*-value |
| --- | --- | --- | --- | --- |
| *Syp* mRNA expression, % | Brown-Forsythe ANOVA  Dunnett’s T3 multiple comparisons test | Group effect  "Young, vehicle vs. Aged, vehicle"  "Young, vehicle vs. Aged, VH-04"  "Aged, vehicle vs. Aged, VH-04" | F*_(2, 5.964)_ = 6.637  *t* = 2.412  *t* = 1.246  *t* = 2.916 | 0.0331  0.1508  0.5316  0.0847 |
